# Danon Disease-Associated LAMP-2 Deficiency Drives Metabolic Signature Indicative of Mitochondrial Aging and Fibrosis in Cardiac Tissue and hiPSC-Derived Cardiomyocytes

**DOI:** 10.3390/jcm9082457

**Published:** 2020-07-31

**Authors:** Giorgia Del Favero, Alois Bonifacio, Teisha J. Rowland, Shanshan Gao, Kunhua Song, Valter Sergo, Eric D. Adler, Luisa Mestroni, Orfeo Sbaizero, Matthew R. G. Taylor

**Affiliations:** 1 Department of Food Chemistry and Toxicology, Faculty of Chemistry, University of Vienna, Währinger Straße, 38–40, 1090 Vienna, Austria; 2 Core Facility Multimodal Imaging University of Vienna. Währinger Straße, 38–40, 1090 Vienna, Austria; 3 Department of Engineering and Architecture, University of Trieste, 34127 Trieste, Italy; abonifacio@units.it (A.B.); sergo@units.it (V.S.); sbaizero@units.it (O.S.); 4 Department of Molecular, Cellular, and Developmental Biology, University of Colorado Boulder, Boulder, CO 80309, USA; teisha.rowland@colorado.edu; 5 Cardiovascular Institute and Adult Medical Genetics, University of Colorado Anschutz Medical Campus, Aurora, CO 80045, USA; SHANSHAN.GAO@CUANSCHUTZ.EDU (S.G.); KUNHUA.SONG@CUANSCHUTZ.EDU (K.S.); Luisa.Mestroni@CUAnschutz.edu (L.M.); 6 Department of Medicine, Division of Cardiology, University of California San Diego, 9500 Gilman Drive, Biomedical Research Facility, La Jolla, CA 92093, USA; eradler@health.ucsd.edu

**Keywords:** Danon disease, LAMP-2 deficiency, cardiac fibrosis, multi-omics profiling, mitochondrial aging phenotype, cell biomechanics

## Abstract

Danon disease is a severe X-linked disorder caused by deficiency of the lysosome-associated membrane protein-2 (LAMP-2). Clinical manifestations are phenotypically diverse and consist of hypertrophic and dilated cardiomyopathies, skeletal myopathy, retinopathy, and intellectual dysfunction. Here, we investigated the metabolic landscape of Danon disease by applying a multi-omics approach and combined structural and functional readouts provided by Raman and atomic force microscopy. Using these tools, Danon patient-derived cardiac tissue, primary fibroblasts, and human induced pluripotent stem cells differentiated into cardiomyocytes (hiPSC-CMs) were analyzed. Metabolic profiling indicated LAMP-2 deficiency promoted a switch toward glycolysis accompanied by rerouting of tryptophan metabolism. Cardiomyocytes’ energetic balance and NAD+/NADH ratio appeared to be maintained despite mitochondrial aging. In turn, metabolic adaption was accompanied by a senescence-associated signature. Similarly, Danon fibroblasts appeared more stress prone and less biomechanically compliant. Overall, shaping of both morphology and metabolism contributed to the loss of cardiac biomechanical competence that characterizes the clinical progression of Danon disease.

## 1. Introduction

Danon disease is a rare, severe X-linked disorder caused by deficiency of lysosome-associated membrane protein-2 (LAMP-2) and characterized by skeletal and cardiac myopathy, retinopathy, and intellectual disability [1]. LAMP-2 is a major glycoprotein component of the lysosomal membrane and is involved in autophagy [2]. Indicative of disrupted autophagy, Danon patients show marked accumulation of late autophagic vacuoles in the heart and skeletal muscle [1,3].

Danon disease is generally more severe in hemizygous males, who show initial symptoms of cardiac involvement at puberty (12 years old on average). These individuals almost invariably develop severe hypertrophic cardiomyopathy and arrhythmia, leading to death or need of heart transplantation (18 years old on average) [4,5]. Mild skeletal myopathy, cognitive defects, and vision problems are also reported, more commonly in males. Heterozygous females typically show signs of disease later in life, presenting with dilated or hypertrophic cardiomyopathy often with cardiac conduction disease and arrhythmia [6].

On the molecular and cellular levels, disrupted autophagy and deregulated mitochondrial functions are evident in Danon disease [7], although it remains unclear how these altered functions cause the severe clinical manifestations. Histological analyses of patient heart tissues reveal severe vacuolization and fibrosis [7]. Patient-derived human induced pluripotent stem cell (hiPSC)-cardiomyocytes (hiPSC-CMs) display impaired mitochondrial function and turnover along with decreased ATP production [7,8,9]. Although normal mitochondrial function is crucial for supporting cellular energetic status, particularly for the high energy demand of muscle cells, how such impaired metabolic competence results in severe cardiac and skeletal muscle impairment [10,11] remains elusive. Because Danon disease is very rare, with <100 clinical reports and <200 families reported in the literature [12], there is poor availability of material for diagnosis and characterization [13]. Hence, research toward the creation of reliable models, such as animal models [14] or iPSC-CMs, enabling the study of this complex pathology even in a limited manner are essential and constantly evolving.

To determine the molecular and cellular mechanisms by which LAMP-2 deficiency results in metabolic changes, here we combined Raman spectroscopy, transcriptomics, and metabolomics approaches on cardiac tissue and cellular models derived from Danon patients. Analyses were performed on cardiac tissue biopsies from Danon patients, as well as from patient-derived hiPSC-CMs. Even with limited patient cardiac tissue, we were able to identify pathways that were similarly impacted in our cellular models, supportive of these pathways being significantly affected in Danon disease. LAMP-2 deficiency appeared to promote specific metabolic switch in cardiomyocytes, possibly adaptive, given that cardiomyocytes have a high metabolic need. The pathway analyses suggest that LAMP-2 deficiency favors glycolysis to oxidative phosphorylation (OXPHOS) and promotes alternative metabolic pathways, such as with the mobilization of tryptophan. This contributes to preserving high nicotinamide adenine dinucleotide (NAD^+^) levels despite mitochondrial aging. Similarly, fibroblasts respond to LAMP-2 deficiency with a stress-associated phenotype pointing toward fibrosis progression. This was accompanied by impairment of biomechanical properties, which retraces the clinical phenotype of Danon disease.

## 2. Experimental Section

### 2.1. Patient Sample Collections

Explanted failing hearts were collected from patients undergoing cardiac transplantation at the University of Colorado Hospital or Children’s Hospital of Colorado under IRB approved cardiac biobank protocols (protocol approved by the University of Colorado Multi-Institutional Review Board). Subjects completed a consent process to donate their native hearts for research purposes. Non-failing left ventricular samples were procured from organ donors whose hearts could not be used for transplants due to size limitations, ABO mismatches, or other factors. For the current study, cardiac tissue from three confirmed Danon subjects was utilized from this tissue bank system. Consent for non-failing samples was obtained through the organ procurement network. At the time of explant, samples of the left-ventricular free wall were immediately sectioned and immersed in liquid nitrogen followed by long-term storage at −80 °C. 15–30 mg of cardiac tissue was sampled for each of the Raman, transcriptomics, and metabolomics tests. All samples came from left-ventricular tissue and were sampled from the left ventricles for analyses. A list of mutations for each myocardial donor is included in Supplementary Table S1. Fibroblast collection and differentiation into hiPSC-CMs were completed under protocols reviewed by the University of Colorado and University of California, San Diego institutional review boards.

### 2.2. Cardiac Tissue Transcriptome Analysis

Frozen cardiac tissue was broken up in liquid nitrogen using mortar and pestle to obtain 2 × 2 × 2 mm size pieces for ribonucleic acid (RNA) analysis. Tissue was placed in TRIzol reagent (Thermo Fisher Scientific, Waltham, MA) and homogenized using a mechanical IKA T25 Ultra-Turaxx homogenizer for 60 s. RNA isolation was performed using mirVana miRNA isolation kits (Thermo Fisher Scientific, Waltham, USA) and enriched for total RNA isolation following the manufacturer’s instructions with an exception of replacing the lysis/binding buffer with TRIzol. All samples were DNase treated using TURBO DNA-free Kit (Thermo Fisher Scientific). PolyA RNA transcripts were enriched from 1µg total RNA using oligo-dT beads and cDNA libraries were constructed using the TruSeq Stranded mRNA Library Prep Kit and protocol from Illumina (Illumina Inc., San Diego, CA). RNA libraries were single-read sequenced on an Illumina HiSeq 2500 for 50 cycles at the University of Colorado Genomics and Microarray Core. Parts of the RNA-seq data presented in Figure 4A were generated in the frame of the study Hashem et al., 2017 [7]; however, they were not included in the publication.

### 2.3. Metabolome Analysis

Metabolomics was completed at the University of Colorado Metabolomics Core. Frozen human cardiac tissues were pulverized with pestle and mortar under liquid nitrogen. 30–50 mg tissue powder was scooped into a microcentrifuge tube and lysed for the following steps. Live hiPSC-CMs and accompanying spent cell culture media were also analyzed as follows. Samples were passed through a C18 reversed-phase column (Phenomenex, Torrance, CA, USA) using an ultrahigh-performance liquid chromatography (UHPLC) system (Vanquish, Themo Fisher). Metabolite assignment, peak integration for relative quantification, and isotopologue distributions in tracing experiments were calculated through the software Maven (Princeton), against the KEGG pathway database and an in-house validated standard library (>650 compounds). Raw data are provided as Supplementary Material 1 (cells) and 2 (medium).

### 2.4. Danon Patient Fibroblast Studies

Primary expanded cultures of human skin fibroblasts were used from a patient with Danon disease and a confirmed LAMP-2 mutation (c.247 C > T; p.Gln83X). Danon disease fibroblasts were compared to those from a wild-type (WT) male control with no known LAMP-2 mutation. Skin biopsies were obtained from subjects following informed consent using 1% local lidocaine anesthesia and 4 mm punch biopsies from subject’s forearms. Samples were place immediately in culture media and cultured by a commercial cytogenetics laboratory. Cells were cultivated in DMEM supplemented with 10% FBS and 1% penicillin/streptomycin. Prior to analysis by atomic force microscopy (AFM), fibroblasts were incubated in serum-free medium for 24 h to induce the formation of autophagic vacuoles and favor glycolysis (i.e., via starvation), typical of Danon disease.

### 2.5. Danon Patient hiPSC-CMs Model

Human skin fibroblasts from Danon and control patients were reprogrammed into hiPSCs as previously described and then differentiated into cardiomyocytes (iPSC-CMs) for study [8,9]. For RNA-seq, undifferentiated hiPSCs were cultured in Geltrex-coated culture dishes with human embryonic stem cell (hESC) medium composed of DMEM/F12 (Cellgro, Mediatech), TGF-β, transferrin, ascorbic acid, selenium sulfate, insulin, and bFGF (R&D Systems), as previously described [7] (PCA plots are provided in Supplementary Figure S1, Danon patients D1−3). For metabolomics, undifferentiated hiPSCs were cultured on hESC-qualified Matrigel (Corning) with mTeSR1 media (STEMCELL Technologies). Differentiation of hiPSCs into cardiomyocytes was carried out as previously described ([15] Danon patient D1). All experiments were done on day 60 of differentiation.

### 2.6. Bioinformatics Pathway Analysis

RNA sequences were analyzed with a bioinformatics pipeline using gSNAP, Cufflinks, and R for sequence alignment and calculation of differential gene expression (CITES). Differential expression was analyzed with ANOVA and corrected for multiple testing by the false-discovery method (FDR ≤ 0.05). Gene ontology analysis was performed with DAVID Bioinformatics Resources (version 6.8, 2020) [16,17]. To this aim, genes differentially regulated between Danon tissue/cells and controls (FDR 0.05 for tissue samples and 0.01 for cell samples) were associated to respective cellular components (CC). Moreover, overexpression enrichment analysis (ORA) was performed with WebGestalt [18]. Overexpressed transcription factors were obtained with the oPOSSUM bioinformatics tool [19,20,21]. Raw data uploaded for the analysis are provided as Supplementary Material 3 and 4.

### 2.7. Raman Spectroscopy

Raman spectra were collected with a Renishaw InVia Raman microscope (Renishaw plc, Wotton-Under-Edge, UK), equipped with a diode laser emitting at 785 nm (300 mW). Tissue sections were investigated by collecting a variable number of spectra (from 400 to 2300) on a regular grid (i.e., Raman maps), with grid step varying from 1.4 um (for high resolution maps) to 90 um (for larger areas). For each spectrum, the acquisition time was 10 s (in the SyncroScan mode of the InVia microscope), and the microscope used was 50× (N.A. 0.75). The calibration of the spectrograph, equipped with a grating of 1200 l/mm, was checked using the 520 cm^−1^ band of a silicon reference sample. The power of the laser at the sample was 150 mW.

All Raman data were pre-processed and analyzed using the R software environment (version 3.6.0) for statistical computing and graphics [22]. For processing spectra and preparing the figures, the *hyperSpec* package was used [23]. Spectra were baseline corrected using a fourth polynomial baseline, using the *baseline* package [24].

### 2.8. Atomic Force Microscopy

Single cells were analysed for assessment of their surface morphology, volume, and elasticity using a Solver-Pro M atomic force microscope (NT-MDT, Moscow, Russia). The setup was equipped with Nova AFM controlling software (NT-MDT, Russia). Details for the morphological analysis (scan time of 10 min for 100 × 100 μm^2^, resolution of 256 × 256 points, pixel size 0.4 × 0.4 μm^2^) and for the force spectroscopy (Young’s modulus calculation) were previously described in detail [25,26]. All experiments were performed on living cells with a liquid scanning AFM set-up. For the analysis we used a cantilever with polystyrene microsphere (Diameter about 10 μm by scanning electron microscopy imaging) coated with a gold layer (Nano and More, Wetzlar, Germany). Resonance frequency (nominally 17 kHz) and spring constant (nominally 0.08 N/m) of the cantilever were checked every day before the beginning of the experiments. For the calibration, values within a 20% variation from those indicated by the supplier were considered acceptable. Measurements were collected in at least three different experimental sessions with different cell preparations of both wild type (controls) and Danon fibroblasts.

### 2.9. Alamar Blue Assay

Alamar Blue experiments were performed using Alamar Blue reagent according to the specifications of the supplier. Briefly, cells were seeded in 96-well plates and Alamar Blue was added to the cell culture medium (dilution 1:10). Cells were then incubated for 4 h at 37 °C, away from light, to allow for the reduction of the dye from metabolically active cells. At the end of the incubation, cell culture supernatant was collected and the fluorescent signal analyzed using a plate reader with 560EX nm/590EM nm filters (EnVision 2401 Multilabel Reader; Perkin Elmer; Waltham, MA, USA). Experimental results are presented as mean of at least two independent cell preparations and measurements performed in six replicates. Distribution of data were considered different with *p* < 0.05 at Student’s *t*-test.

## 3. Results

### 3.1. Transcriptome Analysis

We initially compared Danon patient cardiac tissue with patient hiPSC-CMs to determine how similarly the disease phenotype manifests in the cellular model of patient hiPSC-CMs. RNA sequencing was performed for patient cardiac tissues and patient hiPSC-CMs cells and revealed more than 2000 genes differentially expressed (FDR 0.05) in the hiPSC-CM model and more than 200 in the tissue sample (FDR 0.1), comparing each to relevant healthy controls.

**Figure 1:**
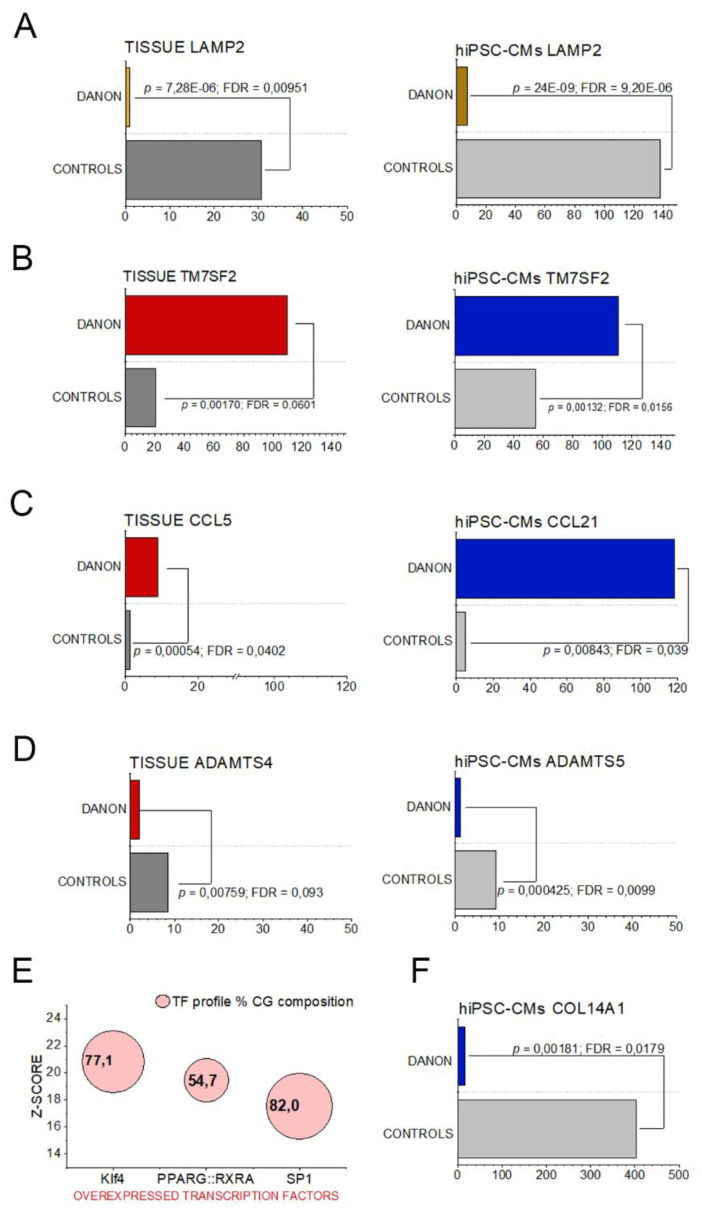
Representative data from the RNA-Seq of cardiac biopsies (indicated as TISSUE, left side) and hiPSC-CMs (right side). Gene expression of LAMP-2 (**A**), Delta(14)-sterol reductase TM7SF2 (**B**), Chemokines CCL21 and CCL5 (**C**), and metalloproteases (ADAMTS4 and ADAMTS5) (**D**). Overexpressed transcription factors identified using oPOSSUM bioinformatics tool with cardiac biopsies (**E**). Expression of collagen gene COL14A1 in hiPSC-CMs (**F**).

Firstly, molecular identity of the pathology could be confirmed since LAMP-2 was, for both sample types, significantly downregulated (Figure 1A). In addition, other similarities could be observed. For example, among the top 10 most abundantly expressed genes in the cardiac tissue was delta (14)-sterol reductase (TM7SF2) and pro-inflammatory chemokine (C-C motif) ligand 5 (CCL5). These regulatory events found a parallel in the hiPSC-CMs with upregulation of TM7SF2 (Figure 1B) and CCL21 (Figure 1C). Similarities could be observed also for downregulated genes, for instance the metalloproteases ADAMTS4 and ADAMTS5 (Figure 1D). oPOSSUM bioinformatics analysis provided an overview of the overexpression of transcription factors primarily associated with the genes regulated in the tissue. This approach highlighted three main transcription factors: KLF4 (Krüppel like factor 4), PPARG (Peroxisome Proliferator-Activated Receptors gamma), and SP1 (Specificity Protein 1, Figure 1E). Intriguingly, these three transcription factors underpin intensive regulation of the morphological and metabolic adaptation strategies. PPARs are crucial regulators of glucose and lipid metabolism [27], SP-1 is involved in response to hypoxia [28,29] and KLF4 sustains inflammation and mitochondrial metabolic adaptation [30,31,32] and promotes senescence [33]. Indeed, mitochondria are central in determining aging phenotypes and associated metabolic states [34]. Particular for the hiPSC-CMs was the regulation of collagen genes (e.g. COL14A1, Figure 1F).

**Figure 2:**
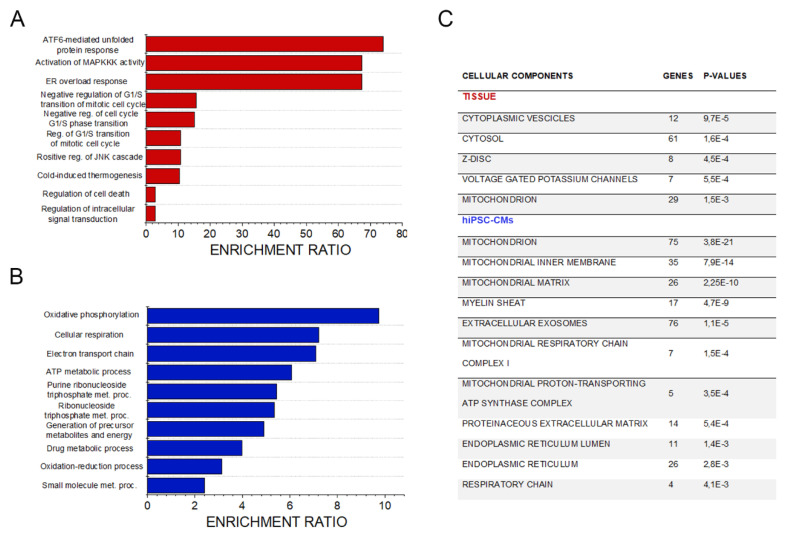
Pathway analysis. Overexpression enrichment analysis of cardiac tissue transcriptome (**A**) and hiPSC-CM transcriptome (**B**). Summary of the cellular components significantly regulated in Danon patients’ derived hiPSC-CMs (FDR 0.01) compared to controls and heart biopsies (FDR 0.05) obtained with the David bioinformatics tool (**C**).

Albeit with some differences, overrepresentation enrichment analysis further suggested the presence of metabolic impairment and a stress management response in both hiPSC-CMs and cardiac tissue samples from Danon disease patients (Figure 2A,B). Tissue analysis highlighted several processes related to the unfolded protein response (UPR [35]; ATF6-mediated unfolded protein response; ER overload response, Figure 2A). These stress response pathways mirror altered autophagy, and play a central role in the regulation of mitochondrial turnover via mitophagy [36]. The UPR can also be triggered by mitochondrial stress and defective mito-nuclear communications [36] with lipid metabolism being a central player of this crosstalk [37]. Mitochondrial respiration, oxidative phosphorylation, and several metabolic processes (ATP metabolic process; purine ribonucleotide triphosphate metabolic process; ribonucleotide triphosphate metabolic process; generation of precursor metabolites and energy) were highlighted in the enrichment analysis of hiPSC-CMs (Figure 2B). More generally, DAVID bioinformatics processing of the data revealed mitochondrial pathways to be common differentially regulated elements of cellular components in the cardiac tissue and hiPSC-CM models compared to healthy controls (Figure 2C). These findings agree with previous data describing differential mitochondrial and oxidative stress pathways being activated in Danon-derived hiPSC-CMs [7,8].

### 3.2. Metabolome Analysis

**Figure 3:**
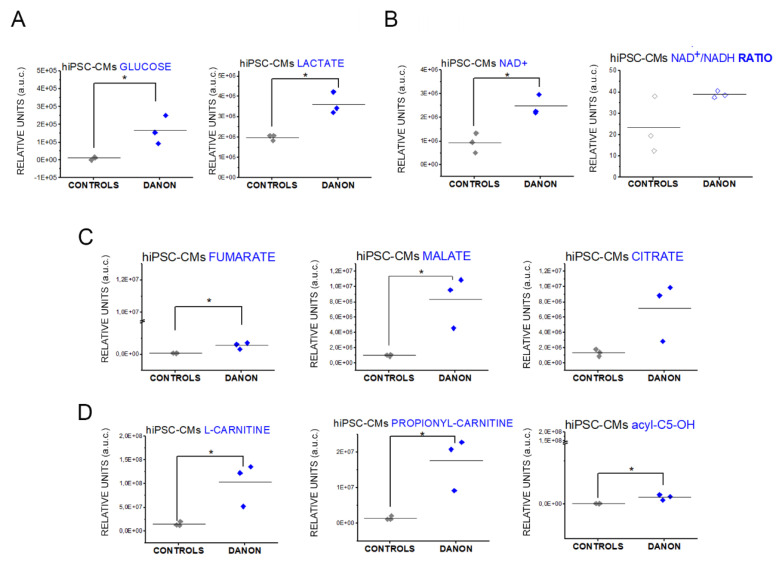
Metabolome profiling of hiPSC-CMs. (**A**) Effect on intracellular glucose level (13.26 fold changes increase) and on intracellular lactate, NAD+ and NAD+/NADH ratio (**B**) TCA cycle ((**C**), fold changes increase: fumarate 7.8; malate 9.19; citrate 6.37) and fatty acids ((**D**), fold changes increase: L-carnitine 9.45; propionyl-carnitine 18.51; acyl-C5-OH 13.06). * Significant difference *p* < 0.05 *t*-test.

Transcriptome data pointed toward a deregulation of mitochondria and stress management capacity. Indeed, mitochondria are central regulators of the metabolic adaptive response [38,39]. Using the metabolomics approach, we observed increased levels of metabolites/decreased clearance in the Danon cells compared to controls. Danon hiPSC-CMs showed an increase in glucose and lactate levels (Figure 3A). Despite the low metabolic competence in terms of ATP generation of these cells (as previously published [7]) the levels of NAD+ remained high and the NAD+/NADH ratio remained comparable to that of control cells (Figure 3B). In addition, other important metabolic intermediates like malate, fumarate, and citrate (TCA cycle, Figure 3C) and carnitine (fatty acids metabolism, Figure 3D) were upregulated.

**Figure 4:**
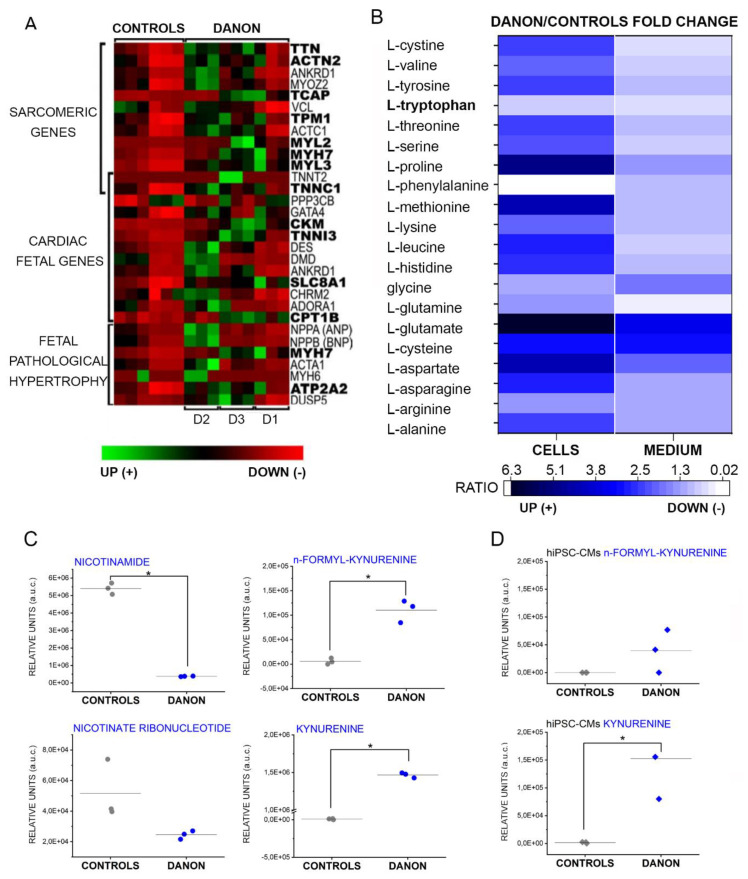
Transcriptomic and metabolic profiling of hiPSC-Cardiomyocytes derived from Danon patients and influence on extracellular environment. (**A**) Transcription levels of structural cardiac elements. (**B**) Amino-acids quantification: fold changes ratios between Danon and controls in the intracellular (cells) and in the extracellular (medium) compartments. (**C**) Relative concentration of nicotinate ribonucleotide and nicotinamide in the culture medium. Quantification of n-formyl-kynurenine and kynurenine in the culture medium (fold changes increase > 25; (**C**)) in the cells (fold changes increase kynurenine > 60; (**D**)) * Significant difference *p* < 0.05 *t*-test.

These findings suggest that Danon hiPSC-CMs perhaps switch toward glycolysis, which might be insufficient to ensure the elevated metabolic need of cardiac cells and contractile activity. In this respect, we also observed consistent increases in gene expression levels of some sarcomeric genes and fetal pathologic hypertrophy-related genes (Figure 4A). Indeed, despite the accumulation of metabolites and increased glucose uptake, these data point toward a general decrease of the contractile capacity of the cells. Of note, these findings were relatively well conserved in the same cell type used for the metabolome profiling (D1) as well as in hiPSC-CMs independently generated from two additional Danon patients (D2 and D3 [7]).

In line, in case of cardiac hypertrophy or senescence, NAD+ pools can be maintained also via secondary routes independent from OXPHOS [40]. Considering the crucial importance of the co-factor, cell supply is ensured via multiple pathways including the potential use of alternative precursors like tryptophan, nicotinamide riboside, nicotinamide, and nicotinic acid [41]. In agreement with this model, we observed on one side a general increase of the amino acids in the cellular fraction (measured as ratio between Danon hiPSC-CMs and controls, Figure 4B), compatible with reduced clearance and/or decrease of the protein synthesis which might originate from ATP deficiency [42]. However, an exception to this trend was tryptophan, which decreased substantially in the Danon hiPSC-CMs (Figure 4B). Tryptophan can also be used as a precursor in the alternative NAD+ biosynthesis [40]. Moreover, we also observed a decrease in the concentration of nicotinate ribonucleotide and nicotinamide in the extracellular medium (Figure 4C) and a significant upregulation of metabolic intermediate kynurenine in the hiPSC-CMs as well as in the extracellular medium (Figure 4C,D).

The analysis of the metabolic regulation of the cardiac biopsies pointed toward a different direction compared to the hiPSC-CMs. In both cases, tryptophan and carnitine-fatty acid metabolism were significantly affected by LAMP-2 deficiency. For kynurenine, we observed a significant increase in the hiPSC-CMs and their medium, however tissue analysis showed a slight, albeit significant decrease. A similar pattern was observed for carnitine and fatty acid metabolism (Table 1 and Table 2). Indeed, the interpretation of the heart samples presented some challenges, potentially because the contribution of the different cell types within the tissue could not be defined (e.g., fibroblasts vs. cardiomyocytes). 

### 3.3. Raman Tissue Analysis

**Figure 5:**
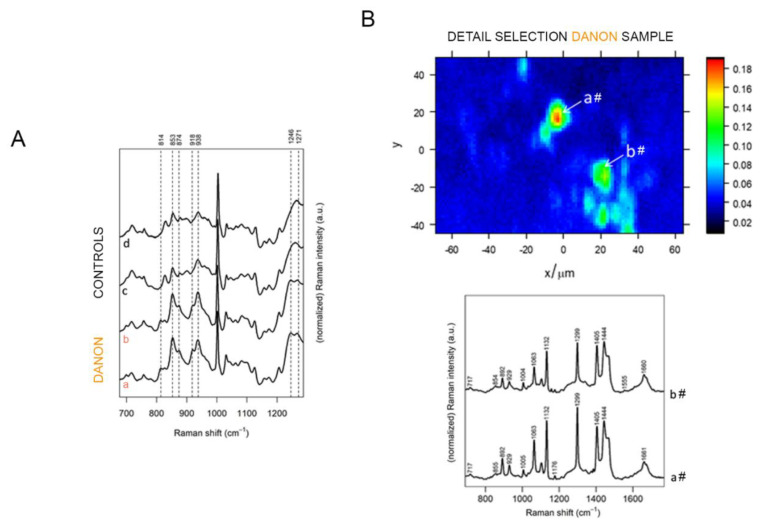
Structural characterization of cardiac tissue. (**A**) Average Raman spectra from tissue sections collected from two Danon patients (a, b, orange) and two controls (c, d, black). The Raman shifts of characteristic collagen bands are shown. Spectra were baseline corrected and intensity normalized with respect to the Phe band at 1002 cm^−1^. (**B**) High-resolution Raman map of a tissue section from a Danon patient and respective spectra of saturated branched fatty acids observed from two different positions (#a, #b). Spectra were baseline corrected and intensity normalized (area normalization). The map depicts the normalized intensity distribution at 1299 cm^−1^, a characteristic Raman shift for saturated lipids.

To evaluate the molecular signature of affected Danon cardiac tissue, and combine it with spatial information about tissue heterogeneity, Raman spectroscopy was performed. This approach allowed us to combine tissue morphological features with correlative label-free chemical speciation. The 700–1300 cm^−1^ region of the average Raman spectra of the tissue sections from two Danon patients and two healthy control subjects are shown in Figure 5A. This region is particularly diagnostic for the presence of collagen, which has a characteristic pattern between 800 and 1000 cm^−1^. Excessive deposition of extracellular matrix (ECM) proteins like collagen plays a central role in cardiac fibrosis and relates to metabolic stress (e.g., senescence, nutritional supply, or diabetes) as well as altered cardiac performance [45,46]. The spectra of Danon samples showed a different pattern with respect to spectra of controls. In particular, the characteristic bands of collagen (marked in Figure 5A with vertical dashed lines) [47], were more intense in the Danon samples than in the controls, indicating a more abundant presence of collagen in the former.

In several locations of a high-resolution Raman map of one of the two specimens collected from Danon patients, the spectra (Figure 5B) are characteristic of saturated branched fatty acids [48]. These lipids were found concentrated in circular areas ranging from about 5 to 10 µm in diameter (Figure 5B). Such lipid vesicles were not observed in a Raman map of a control sample (see Supplementary Figure S2A). Lipid vesicles, however, could not be confirmed in a second sample from a Danon patient (see Supplementary Figure S2B), making the correlation with the Danon phenotype less certain than the observed collagen depositions described above.

### 3.4. Morpho-Mechanical Profiling of Fibroblasts

**Figure 6:**
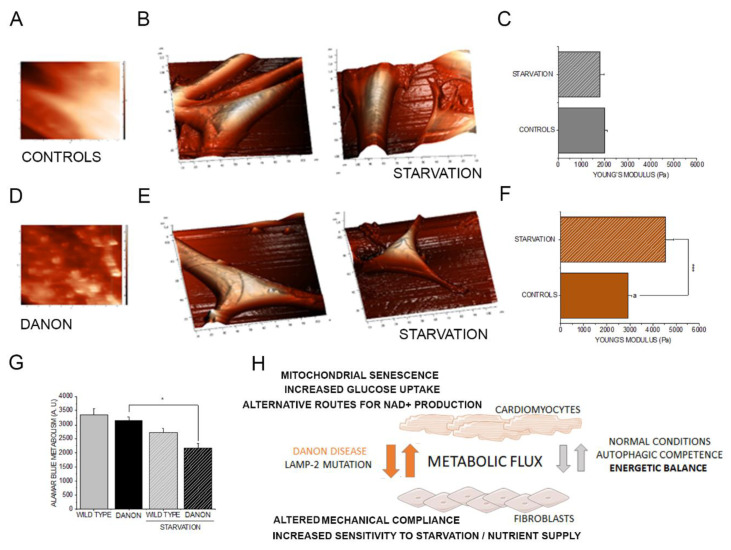
Morphological and functional characterization of fibroblasts via atomic force microscopy. Appearance of primary fibroblasts in complete medium or serum free medium (starvation). Cells after starvation (**A**, Controls; **D**, Danon) and 3D morphological rendering (**B**, Controls; **E**, Danon, scale bars segmentation stands for 10 μm). Measurement of cell stiffness with AFM expressed as Young’s Modulus **C**, Controls; **F**, Danon. (**G**) Alamar Blue assay. Young’s modulus was calculated from *n* > 30 force curves from three cell preparations; a indicates significant difference between Danon and control cells in complete medium Student’s *t*-test *p* < 0.05, * indicate differences between treatment groups * *p* < 0.05, ** *p* < 0.01, and *** *p* < 0.001. (**H**) Schematic representation of the possible crosstalk between cardiomyocytes and fibroblasts in the maintenance of the metabolic need in the heart in health and disease. Graphical elements are taken and adapted from stock images provided by Servier (https://smart.servier.com/smart_image/) under Creative Commons Attribution 3.0 Unported License.

Metabolome profiling and transcriptome analysis of the tissue and of hiPSC-CMs, as well as Raman analysis of cardiac tissues, all suggested the hallmarks of a fibrotic signature associated with the progression of Danon disease. This was indicated by mitochondrial dysfunction, inflammation, altered energy metabolism (Figure 1 and Figure 2), and the structural modifications observed in the tissue (Figure 5). Fibrotic lesions are primarily caused by fibroblasts [45,49] and accompanied by a metabolic switch to activate glycolysis [50]. Increased collagen production, as observed in the Raman maps (Figure 5), and increased stiffness can be attributed to fibroblast activation and sustained fibrosis [51]. Surprisingly, transcriptome analysis of hiPSC-CMs revealed a decrease of the gene expression of collagen production (Supplementary Material 4, Figure 1F). This makes it unlikely that the muscle cells are responsible for the collagen accumulation observed in the tissue, but rather may be calling for an adaptive response to a stiffer environment, which could be created by the fibroblasts. Hence, we tested the hypothesis that Danon patient-derived fibroblasts could be particularly sensitive to metabolic stress and that this could reflect in less compliant mechanical properties. It was previously described that fibroblasts can switch to an anaerobic metabolism (Warburg-like effect) in response to serum deprivation [52], a condition that could resemble the metabolic switch toward glycolysis depicted by the transcriptome and metabolome data for the hiPSC-CMs. Since Danon disease is associated with altered contractile properties and muscular biomechanics, AFM experiments on fibroblasts were performed maintaining the cells in complete medium or in starvation conditions. We compared the response of wild type fibroblasts to LAMP-2 mutants (Danon patient-derived fibroblasts). In line with the clinical phenotype of the disease, Danon fibroblasts were stiffer in comparison to wild type cells and this property was exacerbated in starvation conditions (Figure 6 A–F). Moreover, after serum deprivation, Danon fibroblasts developed a granular appearance (Figure 6A,D), thus reproducing a phenotype in line with that observed in the Raman mapping, and less efficiently metabolized the Alamar Blue reagent (Figure 6G).

## 4. Discussion

The phenotype of Danon disease is associated with severe cardiomyopathy, arrhythmias, and cardiac fibrosis. Here, using a multi-omics approach, we observed a change in cardiomyocyte metabolism characterized by increased glycolysis and alternative NAD+ biosynthesis pathways. This adaptive strategy seems to reflect the attempt to sustain the contractile activity (Figure 4 and [7]), despite altered mitochondrial function, as in the case of senescence and/or impaired mitochondrial turnover (altered mitophagy) [40,53,54]. Since cardiac fibrosis is central in the degeneration of the organ [55], and dysfunctional autophagy has been previously associated with the formation of fibrotic lesions [56], we investigated the molecular events underlying the observed collagen accumulation (Figure 5) via combined transcriptomics–metabolomics analysis of patient cardiac tissue samples as well as cellular patient models.

Hence, considering the limited availability of patient material, we compared global gene expression changes in patient-derived cardiac tissue and hiPSC-CMs and looked for reproducible elements that could describe the disease phenotype. As a first step, we concentrated on the elements of the molecular signature that suggest commonalities between the two sample types. Expression of inflammatory chemokines could be observed in both samples (Figure 1C), with specific upregulation of chemokines CCL21 and CCL5. Intriguingly, CCL5, was previously connected to aging processes [57] and it is a known marker of dexamethasone-resistant chronic inflammation [58]. Also Delta(14)-sterol reductase (Figure 1B, transmembrane 7 superfamily member 2 gene TM7SF2 [59]) was consistently upregulated in cells and tissue samples. Delta(14)-sterol reductase is a key enzyme for cholesterol biosynthesis [60], hence aligning our results with prior histological reports of lipid accumulation in LAMP-2 KO mouse tissue [61] as well as with the Raman spectra reported in this study (Figure 5). This observation, together with the involvement of Peroxisome proliferator-activated receptors (PPRG:RXRA Figure 1E), point toward metabolic changes in relation to loss of LAMP-2. As underpinned by the data processing with the bioinformatics tool oPOSSUM, the regulatory events in Danon patients could be retraced to three main transcription factors (Figure 1E). In addition to the abovementioned PPRG:RXRA, KLF4 was recently described to regulate cardiac mitochondria homeostasis [30] and pro-inflammatory fibrotic injury response [31]. Similarly, SP1 was described as a key player in the induction of cardiac/cardiomyocyte fibrosis [29,62].

Of note, the majority of the cellular components/processes connected to the transcriptome profile of Danon hiPSC-CMs indicate mitochondria and metabolic deregulation (Figure 2C). An exception is the regulation of extracellular matrix and collagen fibrils organization. Interestingly, all genes related to collagen synthesis were downregulated in hiPSC-CMs (Figure 1F and Supplementary Material 4), thus giving an inconsistent picture in comparison to the complete organ pathophysiology characterized by collagen/fibrotic lesions ([4] and Figure 5). We interpreted these data as an adaptive response of the cardiac cells to a less compliant environment created by the onset of the fibrosis. This observation was crucial in guiding toward the description of the individual contribution of cardiomyocytes and fibroblasts.

Indeed, metabolome profiling of hiPSC-CMs revealed distinctive adaptation signatures. We observed an increase of several metabolites (Figure 3 and Figure 4), which could be attributed to elevated biosynthesis or reduced clearance. Likewise, accumulation/reduced clearance of metabolites of the TCA cycle and glycolysis was previously associated with senescent fibroblasts [63]. Intriguingly, the senescent phenotype was also related to increased mitochondrial gene expression/mass [64] and a similar effect could be already described for Danon derived hiPSC-CMs [7]. Similarly, altered mitochondrial distribution was also related to LAMP-2 deficiency in vascular smooth muscle cells [65].

ATP production is constantly required in cardiac mitochondria since the high demand of the tissue does not allow for ATP accumulation/storage [66]. This implies that correct myocardial function largely relies on efficient OXPHOS. However, disease status can be accompanied by an increase of the glycolysis and glucose uptake that seems to be necessary for an early adaptive response to preserve cardiac muscle functionality [67]. Similarly, the heart failure signature is characterized by a decrease of mitochondrial oxidative metabolism with a respective uncoupling of glucose oxidation (decreased) from glycolysis (increased) and associates with cellular acidification and increased ATP consumption for non-contractile purposes [68]. Of note, this metabolic switch retraces a very specific adaptation strategy that has been studied extensively in cancer research, namely the Warburg effect [69]. As originally described by Otto Warburg, cancer cells undergo an energetic/metabolic adaptation from mitochondrial oxidative phosphorylation to glycolysis [39,70,71]. This metabolic switch is generally associated with increased production of lactic acid and, in particular for fibroblasts, collagen secretion [70,72]. Hence, understanding the Danon disease state and metabolic changes could be important also in non-cancer biology. With multiple analogies in the metabolome profile as observed in LAMP-2 deficiency (Figure 3 and Figure 4), a switch toward glycolysis was suggested in the signature of senescent fibroblasts [73] and an increased ECM deposition and fibrosis [74]. Recent reviews connected the Warburg effect to several non-tumor diseases including idiopathic pulmonary fibrosis (IPF), failing heart, cardiac hypertrophy [75], as well as atrial fibrillation [76]. Moreover, proteomics and metabolomics profiling of B-cells isolated from elderly people infer that similar metabolic switching can be related also to the aging process [39]. Taken together, this interpretation would explain the apparent metabolic inefficiency observed in hiPSC-CMs from Danon patients [7] as well as the transcriptomics and metabolomics data of this study.

Of note, it was recently demonstrated that protein turnover follows a strict hierarchical order: proteins constituting the mitochondrial respiratory chain elements are those with the higher recycling speed, especially in Complex I [77]. Hence, accumulation of defective mitochondria as described from altered mitophagy associated with LAMP-2 mutations [7,8] are expected to start a vicious circle, leading to increasingly less efficient ATP production and simultaneously a decreased ability to perform de novo synthesis of mitochondrial subunits necessary of OXPHOS metabolism. In agreement, pathway analysis performed with the RNA-seq data highlighted that mitochondrial-related responses are the most significantly regulated in the hiPSC-CMs (Figure 2). In normal conditions, OXPHOS requires constant transfer of long-chain fatty acids across the inner mitochondrial membrane for subsequent β-oxidation and this essential process is mediated by carnitine [78]. Interestingly, metabolomics data revealed high levels of L-carnitine and propionyl-carnitine in hiPSC-CMs derived from Danon patients in comparison to controls (Figure 3C), but could not be confirmed in the metabolome profile of the tissue (Table 2). This observation might suggest an increase of long-chain fatty acids uptake in the cardiomyocytes driven by the metabolic need, but the inability of the cell to functionally use them due to reduced ATP availability originating from the defective mitochondria. This interpretation could contribute to explaining the lipid accumulation observed in Raman spectra of one cardiac biopsy (Figure 5). Similarly, other metabolic diseases that present as clinical phenotype hypertrophic cardiomyopathy or muscle weakness like VLCADD and LCHAD (very long-/ and long chain-3-hydroxy acyl CoA dehydrogenase deficiency) cause an accumulation of fatty acid metabolites accompanied by mitochondrial morphological alteration, decreased OXPHOS, and ATP and increased glycolysis [79].

Interestingly, despite the alteration of the metabolic efficiency outlined so far, cardiomyocytes preserved high levels of NAD+ and NAD+/NADH ratio (Figure 3B). It was recently described that the nicotinamide riboside kinase (NMRK2) could help to preserve cardiac function and help to maintain NAD+ supply via alternative metabolic routes in chronic heart failure [40]. Similarly, the induction of alternative biosynthetic pathways for NAD+, namely via the administration of the precursor, nicotinamide mononucleotide (NMN), was pursued also in an attempt to reduce the symptoms associated with severe mitochondrial dysfunction in Leigh disease [54]. In our dataset, we observed significant decrease of the nicotinamide (in the extracellular medium, Figure 4C). This suggests a switching in the NAD+ biosynthesis toward pathways bypassing the need of mitochondrial involvement and recalls a senescence/aging phenotype [39,41]. This obviously poses the question of the potential repercussions of the metabolic switch for the resource management of the heart tissue, since it could possibly change metabolites/nutrients availability distribution between the different cell types. In particular for the fatty acid metabolism and for the tryptophan metabolism, the fingerprint of isolated hiPSC-CMs appears different in comparison to the cardiac biopsies, albeit touching the same pathways (Table 1 and Table 2). This can certainly be attributed to tissue heterogeneity as well as to patient inter-individual variability. However, it was previously described that flux of metabolites can be transferred between cell types in close proximity and that fibroblasts can accumulate complementary metabolites to those of cancer cells within the tumor microenvironment [80].

Supportive of the idea that the Danon phenotype could also affect fibroblasts was our observations of decreased mechanical compliance (increased stiffness) in the Danon fibroblasts (Figure 6). Moreover, since cardiac performance reflects the balance between cardiomyocytes and fibroblasts [81], this picture would also fit the data on the fetal pathological hypertrophy presented in Figure 4. Functional impact of fibroblasts on cardiomyocytes is strongly interlinked with senescent phenotype and ‘adult’ fibroblasts were reported to reduce contractile capacity of the muscle cells and promote the deposition of collagen with the creation of a less compliant mechanical environment. On the contrary, fetal fibroblasts could enhance the expression of genes related to cardiomyocyte biomechanical performance [82].

In our experimental conditions, we also observed changes in tryptophan and its metabolites like the *n*-formyl-kynurenine (as observed in Figure 4). These molecules are important not only as metabolites, but also as physiological active molecules. For example, they act as agonists of the Aryl hydrocarbon receptors (AhR) [83]. AhR activation impacts multiple downstream pathways and was recently reported to modulate vascular stiffness in response to aging [84]. This reinforces the interpretation that the metabolic landscape associated with LAMP-2 deficiency could contribute to reducing biomechanical compliance of cardiac tissue via redundant pathways. Likewise, high levels of kynurenine disrupts autophagy and promotes the expression of senescence biomarkers in aged bone marrow mesenchymal stem cells through the activation of the AhR pathway [85], reproducing exactly the molecular events that we could describe in Danon patient derived cells. Overall, our data indicate that the metabolic switch forced by the defective autophagy favors aerobic glycolysis to OXPHOS. In this frame, the interplay between cardiomyocytes and fibroblasts is probably essential in determining the symptomatology associated with Danon disease.

## 5. Conclusions

Data from Danon disease samples obtained with Raman, AFM, transcriptome, and metabolome analyses suggest that LAMP-2 deficiency triggers a profound metabolic switch, possibly associated with rerouting of metabolism toward alternative strategies to maintain NAD+/ NADH ratio. This is necessary to cope with the high energetic need related to cardiac contractile activity and the progressive accumulation of dysfunctional mitochondria. Similarly, LAMP-2 deficiency promotes the onset of fibrosis and a senescence/stress-prone phenotype in fibroblasts (Figure 6H). These biomechanical studies combined with the metabolomics and transcriptomics data outline underlying molecular events leading to the Danon disease phenotype observed in the clinical setting.

## Figures and Tables

**Table 1:** Comparison of the metabolome profile of cardiac tissue in comparison to human induced pluripotent stem cells differentiated into cardiomyocytes (hiPSC-CMs) for indole and tryptophan metabolism. Accession numbers are indicated according the KEGG Metabolome Database [43].

Metabolite		Tissue	hiPSC-CMs	Medium
5-Hydroxyindoleacetate	C05635	0.47	3.33	-
3-Methyleneoxindole	C02796	1.33	-	-
Indole	C00463	3.60	0.94	1.06
Indolepyruvate	C00331	0.95	-	-
Kynurenine	C00328	0.30	69.01	138.36
N-formyl kynurenine	C02700	0.40	-	26.26
Anthranilate	C00108	0.39	-	-
Picolinic acid	C10164	0.52	-	-
g-Oxalo-crotonate	C03453	3.11	-	-
		**Fold changes to controls**		

Colored fields significant difference *p* < 0.05, orange upregulation, blue downregulation.

**Table 2:** Comparison of the metabolome profile of cardiac tissue in comparison to hiPSC-CMs for carnitine and fatty acid metabolism. Accession numbers are indicated according the KEGG [43] or the Human Metabolome Database (HMDB [44]).

Metabolite		Tissue	hiPSC-CMs	Medium
L-carnitine	C00318	0.17	9.45	1.11
Acetyl-carnitine	C02571	0.27	-	-
Propionyl-carnitine	C03017	0.32	18.51	6.66
Butanoyl-l-carnitine	C02862	0.96	5.16	2.04
Acyl-C4-OH	HMDB13127	2.65	1.91	2.84
Acyl-C4-DC	HMDB13133	0.17	1.73	-
Acyl-C5	C20826	0.11	-	-
Acyl-C5:1	HMDB02366	0.24	-	-
Acyl-C5-OH	ac107	0.15	13.06	2.83
		**Fold changes to controls**		

Colored fields significant difference *p* < 0.05, orange upregulation, blue downregulation.

## Supplementary Materials

The following are available online at https://www.mdpi.com/2077-0383/9/8/2457/s1, Figure S1: Principal Component Analysis (PCA) Plot of RNA-Seq Data of hiPSC-CM Samples, Figure S2: High-resolution Raman map of a tissue section from a control sample (A) or non-responsive Danon patient (B), Table S1: Amino acid LAMP-2 mutation list.
