# Supplementary material for: Danon Disease-Associated LAMP-2 Deficiency Drives Metabolic Signature Indicative of Mitochondrial Aging and Fibrosis in Cardiac Tissue and hiPSC-Derived Cardiomyocytes
Source: J Clin Med. 2020 Jul 31;9(8):2457. doi: 10.3390/jcm9082457 (PMC7465084; doi:10.3390/jcm9082457)
Supplement: Supplementary file 1 [file jcm-09-02457-s001.zip › Supplementary Material Del Favero MDPI_JCM/Supplementary Material 1_Metabolome Analysis CELLS.pdf]

hiPS-CM Metabolome analysis CELLS

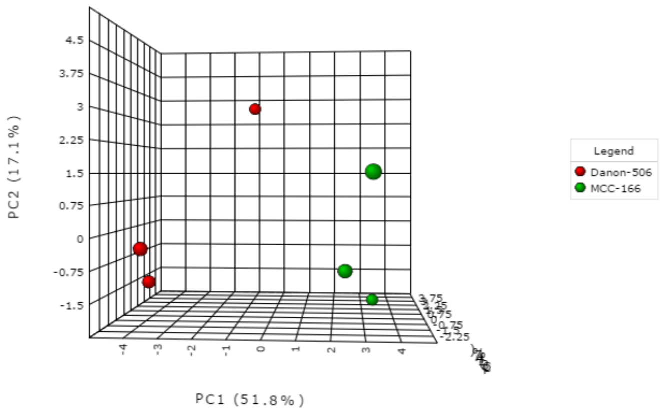

Principal Component Analysis

RAW DATA

							MCC-166_CONTROLS			Danon-506			MEDIAN		FOLD CHANGE	p-value
N.	compound	CmpdID	Pathway	parent	medRt	Polarity	M1	M2	M3	D1	D2	D3	M	D	Danon/MCC	M
1	L-alanine	C00041	Amino acids	90,0559	1,20	+	9,51E+05	8,01E+05	7,97E+05	1,73E+06	2,13E+06	2,03E+06	8,01E+05	2,03E+06	2,53	0,00
2	L-arginine	C00062	Amino acids	175,1194	1,18	+	6,99E+06	6,40E+06	6,81E+06	9,68E+06	7,09E+06	1,02E+07	6,81E+06	9,68E+06	1,42	0,08
3	L-asparagine	C00152	Amino acids	133,0612	1,20	+	2,18E+05	3,36E+05	2,89E+05	5,42E+05	8,44E+05	1,07E+06	2,89E+05	8,44E+05	2,92	0,03
4	L-aspartate	C00049	Amino acids	134,0452	1,22	+	3,76E+05	4,09E+05	3,52E+05	1,59E+06	1,55E+06	1,66E+06	3,76E+05	1,59E+06	4,23	0,00
5	L-cysteine	C00097	Amino acids	122,0276	1,23	+	2,23E+05	3,27E+05	3,45E+05	6,99E+05	9,73E+05	1,13E+06	3,27E+05	9,73E+05	2,97	0,01
6	L-glutamate	C00025	Amino acids	148,0608	1,21	+	1,14E+07	1,60E+07	1,92E+07	9,04E+07	1,01E+08	1,29E+08	1,60E+07	1,01E+08	6,31	0,00
7	L-glutamine	C00064	Amino acids	147,0768	1,20	+	9,50E+05	1,71E+06	1,85E+06	1,49E+06	2,46E+06	5,13E+06	1,71E+06	2,46E+06	1,44	0,25
8	glycine	C00037	Amino acids	76,0403	1,22	+	4,10E+06	3,68E+06	4,16E+06	3,73E+06	4,63E+06	4,90E+06	4,10E+06	4,63E+06	1,13	0,32
9	L-histidine	C00135	Amino acids	156,0771	1,11	+	2,53E+05	3,06E+05	3,23E+05	7,20E+05	1,01E+06	8,03E+05	3,06E+05	8,03E+05	2,62	0,00
10	L-leucine	C00123	Amino acids	132,1023	1,24	+	9,71E+06	1,44E+07	1,07E+07	2,66E+07	3,12E+07	3,01E+07	1,07E+07	3,01E+07	2,81	0,00
11	L-lysine	C00047	Amino acids	147,1131	1,10	+	1,14E+06	1,38E+06	1,34E+06	2,71E+06	2,90E+06	2,69E+06	1,34E+06	2,71E+06	2,02	0,00

12	L-methionine	C00073	Amino acids	150,0577	1,24	+	1,93E+05	2,74E+05	5,96E+05	1,19E+06	1,32E+06	1,20E+06	2,74E+05	1,20E+06	4,39	0,00
13	L-phenylalanine	C00079	Amino acids	166,0861	1,24	+	8,45E+05	1,29E+06	3,49E+04	3,07E+04	2,92E+06	2,51E+04	8,45E+05	3,07E+04	0,04	0,81
14	L-proline	C00148	Amino acids	116,0712	1,23	+	8,57E+06	2,13E+07	1,82E+07	4,79E+07	8,99E+07	1,00E+08	1,82E+07	8,99E+07	4,94	0,02
15	L-serine	C00065	Amino acids	106,0506	1,20	+	2,36E+05	1,97E+05	2,49E+05	4,86E+05	5,29E+05	6,40E+05	2,36E+05	5,29E+05	2,25	0,00
16	L-threonine	C00188	Amino acids	120,0661	1,21	+	3,18E+05	4,06E+05	5,02E+05	8,00E+05	9,71E+05	1,13E+06	4,06E+05	9,71E+05	2,39	0,01
17	L-tryptophan	C00078	Amino acids	205,0976	1,34	+	6,28E+05	2,08E+05	1,05E+05	8,51E+04	5,39E+05	1,38E+05	2,08E+05	1,38E+05	0,66	0,79
18	L-tyrosine	C00082	Amino acids	182,0815	1,24	+	1,56E+06	2,38E+06	2,04E+06	4,86E+06	4,73E+06	4,99E+06	2,04E+06	4,86E+06	2,38	0,00
19	L-valine	C00183	Amino acids	118,0869	1,24	+	4,93E+06	5,74E+06	7,48E+06	1,13E+07	1,08E+07	1,18E+07	5,74E+06	1,13E+07	1,97	0,00
20	L-cystine	C00491	Amino acids	241,0310	1,24	+	2,14E+04	1,61E+04	1,53E+04	3,73E+04	6,91E+04	3,90E+04	1,61E+04	3,90E+04	2,42	0,04
21	ADP	C00008	Nucleotides	426,0232	1,48	-	3,68E+05	3,48E+05	2,63E+05	9,62E+05	4,42E+06	4,23E+06	3,48E+05	4,23E+06	12,18	0,06
22	AMP	C00020	Nucleotides	348,0710	1,26	+	2,43E+06	1,04E+06	1,01E+06	4,44E+06	1,78E+07	1,29E+07	1,04E+06	1,29E+07	12,37	0,06
23	dAMP	C00360	Nucleotides	330,0548	1,26	-	0,00E+00	1,04E+04	1,00E+04	2,91E+04	2,98E+04	3,02E+04	1,00E+04	2,98E+04	2,97	0,00
24	Adenosine	C00212	Nucleotides	268,1044	1,27	+	3,04E+04	2,51E+04	4,71E+04	2,98E+04	1,46E+06	6,51E+05	3,04E+04	6,51E+05	21,45	0,18
25	Adenine	C00147	Nucleotides	136,0622	1,24	+	1,59E+06	1,58E+06	1,50E+06	1,67E+06	1,86E+06	1,86E+06	1,58E+06	1,86E+06	1,17	0,03
26	GDP	C00035	Nucleotides	442,0178	1,94	-	2,33E+04	2,70E+03	0,00E+00	3,44E+03	2,09E+05	2,43E+05	2,70E+03	2,09E+05	77,37	0,13
27	GMP	C00144	Nucleotides	364,0659	1,25	+	4,00E+05	2,41E+05	4,58E+05	4,34E+05	1,66E+06	9,90E+05	4,00E+05	9,90E+05	2,48	0,14
28	Guanosine	C00387	Nucleotides	284,0995	1,25	+	1,28E+04	3,21E+04	1,07E+05	2,45E+05	1,34E+05	1,26E+05	3,21E+04	1,34E+05	4,17	0,07
29	Guanine	C00242	Nucleotides	152,0571	1,25	+	1,21E+05	1,25E+05	1,89E+05	5,76E+05	3,79E+05	3,25E+05	1,25E+05	3,79E+05	3,04	0,02
30	CDP	C00112	Nucleotides	402,0115	1,34	-	3,32E+04	1,67E+04	1,82E+04	4,66E+04	1,31E+05	1,45E+05	1,82E+04	1,31E+05	7,19	0,05
31	CMP	C00055	Nucleotides	324,0599	1,25	+	1,98E+05	1,43E+05	2,39E+05	3,98E+05	4,12E+05	5,36E+05	1,98E+05	4,12E+05	2,08	0,01
32	Cytidine	C00475	Nucleotides	244,0932	1,24	+	3,72E+04	2,74E+04	1,14E+05	1,79E+05	5,71E+05	3,61E+05	3,72E+04	3,61E+05	9,73	0,06
33	Cytosine	C00380	Nucleotides	112,0513	1,24	+	9,74E+04	9,20E+04	1,74E+05	2,00E+05	4,55E+05	4,45E+05	9,74E+04	4,45E+05	4,56	0,05
34	Thymine	C00178	Nucleotides	127,0507	1,23	+	2,81E+04	5,38E+04	7,01E+04	5,27E+04	1,05E+05	1,16E+05	5,38E+04	1,05E+05	1,95	0,15
35	UDP	C00015	Nucleotides	405,0103	1,79	+	4,32E+05	2,71E+05	2,22E+05	8,60E+05	2,08E+06	2,26E+06	2,71E+05	2,08E+06	7,67	0,03
36	IMP	C00130	Nucleotides	349,0552	1,25	+	2,85E+05	1,04E+06	1,46E+06	4,63E+05	3,96E+05	2,96E+05	1,04E+06	3,96E+05	0,38	0,19

37	Inosine	C00294	Nucleotides	269,0885	1,25	+	3,96E+04	1,16E+05	2,44E+05	3,75E+05	1,29E+05	1,57E+05	1,16E+05	1,57E+05	1,34	0,43
38	Hypoxanthine	C00262	Nucleotides	137,0462	1,25	+	5,76E+05	1,20E+06	1,90E+06	2,87E+06	1,18E+06	1,41E+06	1,20E+06	1,41E+06	1,17	0,42
39	Xanthine	C00385	Nucleotides	151,0253	1,59	-	4,87E+03	4,77E+03	1,17E+04	2,61E+03	2,76E+03	5,49E+03	4,87E+03	2,76E+03	0,57	0,23
40	Allantoate	C00499	Nucleotides	177,0613	1,13	+	1,32E+05	1,55E+05	1,05E+05	3,17E+05	1,23E+05	1,44E+05	1,32E+05	1,44E+05	1,10	0,37
41	(S)(+)-Allantoin	C02350	Nucleotides	157,0358	1,24	-	4,38E+04	3,81E+04	7,48E+04	1,30E+05	8,66E+04	9,47E+04	4,38E+04	9,47E+04	2,16	0,04
42	5-Hydroxyisourate	C11821	Nucleotides	185,0302	1,08	+	6,69E+03	5,41E+03	1,06E+04	2,31E+04	1,80E+04	2,14E+04	6,69E+03	2,14E+04	3,21	0,00
43	Urate	C00366	Nucleotides	167,0215	1,24	-	1,03E+05	1,39E+05	2,15E+05	2,69E+05	2,19E+05	3,08E+05	1,39E+05	2,69E+05	1,94	0,05
44	5-6-Dihydrothymine	C00906	Nucleotides	129,0664	1,23	+	3,04E+05	6,31E+05	5,72E+05	2,28E+06	2,89E+06	4,03E+06	5,72E+05	2,89E+06	5,05	0,01
45	Pyridoxal	C00250	Nucleotides	168,0659	1,24	+	4,32E+04	2,38E+04	3,15E+04	7,17E+04	8,92E+04	6,68E+04	3,15E+04	7,17E+04	2,27	0,01
46	4-Pyridoxate	C00847	Nucleotides	184,0601	1,26	+	0,00E+00	3,07E+03	1,82E+04	0,00E+00	5,19E+04	3,41E+04	3,07E+03	3,41E+04	11,11	0,25
47	Pyridoxamine 5'-phosphate	C00647	Nucleotides	249,0624	1,24	+	8,28E+03	3,22E+03	3,61E+03	4,03E+03	3,15E+03	4,13E+03	3,61E+03	4,03E+03	1,11	0,49
48	Nicotinamide	C00153	Nucleotides	123,0558	1,25	+	1,32E+06	2,10E+06	1,78E+06	2,00E+06	2,92E+06	2,80E+06	1,78E+06	2,80E+06	1,57	0,08
49	Nicotinate ribonucleotide	C01185	Nucleotides	335,0457	1,13	-	2,31E+03	0,00E+00	0,00E+00	8,89E+02	0,00E+00	0,00E+00	0,00E+00	0,00E+00	#DIV/0!	0,60
50	UDP-glucose	C00029	Nucleotides	567,0635	1,76	+	1,42E+03	3,51E+03	3,73E+03	2,19E+04	4,88E+04	6,06E+04	3,51E+03	4,88E+04	13,91	0,02
51	ADP-D-ribose	C01882	Nucleotides	560,0803	1,33	+	2,56E+04	3,01E+04	2,74E+04	3,04E+03	2,43E+03	3,02E+03	2,74E+04	3,02E+03	0,11	0,00
52	NAD+	C00003	Nucleotides	664,1177	1,24	+	5,05E+05	1,33E+06	9,56E+05	2,19E+06	2,96E+06	2,26E+06	9,56E+05	2,26E+06	2,36	0,01
53	NADH	C00004	Nucleotides	666,1332	1,86	+	4,05E+04	3,50E+04	4,88E+04	5,42E+04	7,91E+04	5,85E+04	4,05E+04	5,85E+04	1,44	0,06
54	Phosphate	C00009	Phosphates	98,9850	1,25	+	9,46E+07	1,01E+08	9,56E+07	5,79E+07	7,08E+07	5,86E+07	9,56E+07	5,86E+07	0,61	0,00
55	Diphosphate	C00013	Phosphates	176,9353	1,27	-	6,31E+06	5,73E+06	5,49E+06	4,11E+06	5,51E+06	4,93E+06	5,73E+06	4,93E+06	0,86	0,10
56	D-Glucose	C00031	Glycolysis	179,0559	1,18	-	1,86E+04	1,17E+04	0,00E+00	9,24E+04	1,55E+05	2,50E+05	1,17E+04	1,55E+05	13,26	0,03
57	D-Glucose 6-phosphate	C02965	Glycolysis	259,0228	1,27	-	1,10E+05	1,08E+05	1,36E+05	2,11E+05	1,72E+05	1,98E+05	1,10E+05	1,98E+05	1,79	0,01
58	D-Fructose 1-6-bisphosphate	C00354	Glycolysis	338,9892	1,64	-	4,22E+06	4,15E+06	8,67E+06	1,28E+07	2,50E+06	6,83E+06	4,22E+06	6,83E+06	1,62	0,64
59	D-Glyceraldehyde 3-phosphate/Glycerone phosphate	C00118	Glycolysis	168,9891	1,27	-	1,74E+05	1,67E+05	2,30E+05	3,12E+05	1,55E+05	1,93E+05	1,74E+05	1,93E+05	1,11	0,59
60	2/3-Phospho-D-glycerate	C00631	Glycolysis	184,9851	1,31	-	2,31E+06	2,13E+06	1,65E+06	2,48E+06	1,18E+06	1,24E+06	2,13E+06	1,24E+06	0,58	0,44
61	Phosphoenolpyruvate	C00074	Glycolysis	166,9744	1,40	-	1,39E+05	1,69E+05	1,65E+05	1,68E+05	7,80E+04	8,20E+04	1,65E+05	8,20E+04	0,50	0,19

62	Pyruvate	C00022	Glycolysis	87,0074	1,36	-	0,00E+00	0,00E+00	4,79E+05	0,00E+00	5,98E+05	5,03E+05	0,00E+00	5,03E+05	#DIV/0!	0,45
63	Lactate	C01432	Glycolysis	89,0230	1,28	-	1,84E+06	2,08E+06	2,06E+06	3,22E+06	3,42E+06	4,22E+06	2,06E+06	3,42E+06	1,66	0,01
64	Maltose	C00208	Other sugars	343,1236	1,17	+	1,70E+05	1,64E+05	2,39E+05	3,70E+05	2,36E+05	2,24E+05	1,70E+05	2,36E+05	1,38	0,18
65	Citrate	C00158	TCA cycle	191,0194	1,26	-	8,74E+05	1,39E+06	1,78E+06	2,86E+06	8,84E+06	9,88E+06	1,39E+06	8,84E+06	6,37	0,06
66	2-Oxoglutarate	C00026	TCA cycle	145,0143	1,28	-	4,49E+04	4,88E+04	3,80E+04	2,92E+05	1,47E+06	1,03E+06	4,49E+04	1,03E+06	22,89	0,06
67	Succinate	C00042	TCA cycle	117,0181	1,29	-	9,68E+04	1,06E+05	1,28E+05	1,57E+05	2,11E+05	1,62E+05	1,06E+05	1,62E+05	1,53	0,03
68	Fumarate	C00122	TCA cycle	115,0025	1,29	-	6,91E+04	8,25E+04	7,88E+04	3,36E+05	6,15E+05	7,12E+05	7,88E+04	6,15E+05	7,80	0,01
69	Malate	C00149	TCA cycle	133,0131	1,28	-	8,74E+05	1,09E+06	1,04E+06	4,55E+06	9,58E+06	1,09E+07	1,04E+06	9,58E+06	9,19	0,02
70	2-Hydroxyglutarate/Citramalate	C02630	Alternative Carboxylic acids	147,0291	1,25	-	1,01E+05	1,47E+05	1,19E+05	1,01E+06	1,69E+06	2,00E+06	1,19E+05	1,69E+06	14,24	0,01
71	6-Phospho-D-gluconate	C00345	Pentose Phosphate Pathway	275,0180	1,29	-	1,73E+04	2,36E+04	2,49E+04	4,37E+04	4,37E+04	5,75E+04	2,36E+04	4,37E+04	1,85	0,01
72	D-Erythrose 4-phosphate	C00279	Pentose Phosphate Pathway	199,0009	1,25	-	2,80E+04	1,02E+05	1,04E+05	7,03E+04	8,13E+04	8,78E+04	1,02E+05	8,13E+04	0,80	0,94
73	Sedoheptulose 7-phosphate	C05382	Pentose Phosphate Pathway	289,0346	1,28	-	1,90E+03	0,00E+00	2,56E+03	4,89E+03	2,47E+04	2,95E+04	1,90E+03	2,47E+04	13,02	0,07
74	Pentose phosphates (isobars)	C00199	Pentose Phosphate Pathway	229,0117	1,26	-	8,46E+04	1,02E+05	1,06E+05	2,56E+05	1,48E+05	1,41E+05	1,02E+05	1,48E+05	1,45	0,09
75	Glutathione	C00051	GSH homeostasis	308,0916	1,25	+	6,06E+06	1,17E+07	1,16E+07	2,21E+07	3,69E+07	3,07E+07	1,16E+07	3,07E+07	2,65	0,01
76	5-Oxoproline	C01879	GSH homeostasis	130,0503	1,23	+	3,15E+06	4,91E+06	5,22E+06	2,34E+07	2,71E+07	3,65E+07	4,91E+06	2,71E+07	5,51	0,00
77	S-Glutathionyl-L-cysteine	C05526	GSH homeostasis	427,0963	1,23	+	1,58E+04	9,53E+03	4,02E+04	5,90E+04	1,37E+04	4,61E+04	1,58E+04	4,61E+04	2,91	0,34
78	Cys-Gly	C01419	GSH homeostasis	179,0489	1,24	+	1,07E+05	1,64E+05	1,59E+05	5,63E+05	8,53E+05	9,77E+05	1,59E+05	8,53E+05	5,37	0,01
79	Ascorbate	C00072	GSH homeostasis	175,0261	1,23	-	0,00E+00	0,00E+00	3,66E+04	3,31E+04	3,49E+04	4,39E+04	0,00E+00	3,49E+04	#DIV/0!	0,12
80	Dehydroascorbate	C05422	GSH homeostasis	173,0088	1,25	-	1,62E+04	2,40E+04	3,83E+04	5,28E+04	1,49E+05	1,42E+05	2,40E+04	1,42E+05	5,93	0,05
81	gamma-L-Glutamyl-L-cysteine	C00669	Gamma-glutamyls	251,0701	1,25	+	1,16E+04	3,35E+04	1,53E+04	1,12E+05	9,24E+04	8,10E+04	1,53E+04	9,24E+04	6,03	0,00
82	gamma-L-Glutamyl-D-alanine	C03738	Gamma-glutamyls	219,0983	1,25	+	4,26E+04	5,66E+04	5,39E+04	1,56E+05	1,38E+05	1,30E+05	5,39E+04	1,38E+05	2,57	0,00
83	gamma-Glutamyl-gamma-aminobutyrate	C15767	Gamma-glutamyls	233,1143	1,25	+	2,15E+04	3,44E+04	9,90E+03	9,70E+04	1,47E+05	1,18E+05	2,15E+04	1,18E+05	5,50	0,00
84	gamma-L-Glutamylputrescine	C15699	Gamma-glutamyls	218,1506	1,09	+	3,69E+04	5,02E+04	3,07E+04	4,43E+04	4,62E+04	2,99E+04	3,69E+04	4,43E+04	1,20	0,92
85	(5-L-Glutamyl)-L-glutamine	C05283	Gamma-glutamyls	276,1196	1,23	+	2,77E+04	5,32E+04	6,62E+04	1,40E+05	2,25E+05	4,37E+05	5,32E+04	2,25E+05	4,23	0,07
86	Dimethylglycine	C01026	Serine biosynthesis and one-carbon metabolism	104,0714	1,29	+	3,00E+05	4,72E+05	1,13E+06	3,33E+05	3,40E+05	3,31E+05	4,72E+05	3,33E+05	0,71	0,30

87	Phosphoserine	C02532	Serine biosynthesis and one-carbon metabolism	186,0166	1,21	+	1,40E+04	5,74E+04	5,11E+04	4,16E+05	8,30E+05	7,86E+05	5,11E+04	7,86E+05	15,37	0,01
88	S-Adenosyl-L-homocysteine	C00021	Serine biosynthesis and one-carbon metabolism	385,1293	1,25	+	7,69E+03	3,56E+03	1,11E+04	9,15E+04	1,14E+05	1,03E+05	7,69E+03	1,03E+05	13,41	0,00
89	S-Adenosyl-L-methionine	C00019	Serine biosynthesis and one-carbon metabolism	399,1451	1,20	+	1,45E+04	5,82E+03	1,99E+04	2,43E+04	3,03E+04	4,01E+04	1,45E+04	3,03E+04	2,09	0,04
90	3-Phosphonopyruvate	C02798	Serine biosynthesis and one-carbon metabolism	168,9901	1,40	+	1,64E+05	1,89E+05	1,96E+05	2,01E+05	8,67E+04	7,75E+04	1,89E+05	8,67E+04	0,46	0,20
91	Ornithine	C01602	Urea cycle	133,0976	1,10	+	3,14E+05	3,42E+05	2,93E+05	8,77E+05	4,24E+05	3,96E+05	3,14E+05	4,24E+05	1,35	0,19
92	L-Citrulline	C00327	Urea cycle	176,1035	1,21	+	2,37E+04	1,81E+04	5,53E+04	8,03E+04	3,55E+04	6,62E+04	2,37E+04	6,62E+04	2,80	0,18
93	Argininosuccinate	C03406	Urea cycle	291,1303	1,20	+	0,00E+00	0,00E+00	3,28E+03	4,10E+04	4,62E+04	9,20E+04	0,00E+00	4,62E+04	#DIV/0!	0,02
94	Putrescine	C00134	Polyamines	89,1081	1,15	+	0,00E+00	0,00E+00	6,48E+05	3,16E+05	1,43E+06	0,00E+00	0,00E+00	3,16E+05	#DIV/0!	0,49
95	Spermidine	C00315	Polyamines	146,1655	1,13	+	3,31E+05	5,94E+05	1,23E+06	3,81E+06	1,04E+07	1,07E+07	5,94E+05	1,04E+07	17,48	0,03
96	Spermine	C00750	Polyamines	203,2234	1,15	+	4,81E+04	4,71E+04	1,40E+05	1,04E+05	2,49E+05	2,26E+05	4,81E+04	2,26E+05	4,71	0,10
97	N-Acetylneuraminate	C00270	Aminosugars	310,1138	1,21	+	7,46E+04	8,75E+04	1,11E+05	8,24E+05	7,05E+05	6,86E+05	8,75E+04	7,05E+05	8,07	0,00
98	CMP-N-acetylneuraminate	C00128	Aminosugars	615,1558	1,24	+	5,68E+04	8,19E+04	9,24E+04	3,17E+05	2,86E+05	2,22E+05	8,19E+04	2,86E+05	3,49	0,00
99	beta-L-Arabinose 1-phosphate	C03906	Aminosugars	231,0281	1,63	+	0,00E+00	1,50E+03	0,00E+00	1,59E+04	3,24E+04	1,58E+04	0,00E+00	1,59E+04	#DIV/0!	0,02
100	5-Guanidino-2-oxopentanoate	C03771	Arginine and proline metabolism	174,0879	1,24	+	0,00E+00	0,00E+00	0,00E+00	4,38E+03	1,48E+04	9,26E+03	0,00E+00	9,26E+03	#DIV/0!	0,03
101	Phosphocreatine	C02305	Arginine and proline metabolism	212,0434	1,27	+	2,88E+05	5,92E+05	4,30E+05	3,68E+05	2,09E+05	4,10E+05	4,30E+05	3,68E+05	0,86	0,37
102	Creatine	C00300	Arginine and proline metabolism	132,0772	1,23	+	6,36E+06	8,44E+06	1,21E+07	1,61E+07	1,95E+07	2,94E+07	8,44E+06	1,95E+07	2,31	0,04
103	Creatinine	C00791	Arginine and proline metabolism	114,0668	1,22	+	1,91E+05	2,13E+05	3,86E+05	7,28E+05	6,38E+05	8,56E+05	2,13E+05	7,28E+05	3,42	0,01
104	N-Acetyl-L-citrulline	C15532	Arginine and proline metabolism	218,1140	1,22	+	0,00E+00	0,00E+00	4,98E+03	2,09E+04	7,46E+03	2,38E+04	0,00E+00	2,09E+04	#DIV/0!	0,04
105	N-Acetylornithine	C00437	Arginine and proline metabolism	175,1083	1,25	+	7,12E+04	7,74E+04	6,84E+04	2,56E+05	2,14E+05	2,70E+05	7,12E+04	2,56E+05	3,59	0,00
106	Guanidinoacetate	C00581	Arginine and proline metabolism	118,0617	1,22	+	6,05E+03	2,81E+04	2,92E+04	1,03E+04	8,56E+04	1,28E+05	2,81E+04	8,56E+04	3,05	0,20
107	trans-4-Hydroxy-L-proline	C01157	Arginine and proline metabolism	132,0660	1,21	+	8,32E+05	9,16E+05	1,10E+06	1,99E+06	2,56E+06	3,40E+06	9,16E+05	2,56E+06	2,79	0,02
108	5-Aminopentanoate	C00431	Arginine and proline metabolism	118,0869	1,64	+	2,38E+06	0,00E+00	3,91E+06	2,12E+06	2,34E+06	3,89E+06	2,38E+06	2,34E+06	0,98	0,61
109	L-1-Pyrroline-3-hydroxy-5-carboxylate	C04281	Arginine and proline metabolism	130,0503	1,80	+	3,25E+05	3,86E+05	3,58E+05	9,09E+05	1,03E+06	1,08E+06	3,58E+05	1,03E+06	2,87	0,00
110	Pantothenol	C00864	Panthothenate metabolism	220,1184	1,33	+	5,95E+04	9,78E+04	1,03E+05	2,61E+05	7,29E+05	5,05E+05	9,78E+04	5,05E+05	5,16	0,04

111	Pantetheine	C00831	Panthothenate metabolism	279,1355	1,26	+	4,19E+04	0,00E+00	0,00E+00	0,00E+00	3,03E+04	6,26E+04	0,00E+00	3,03E+04	#DIV/0!	0,50
112	Taurine	C00245	Sulfur metabolism	126,0224	1,25	+	1,75E+05	3,58E+05	3,38E+05	3,44E+05	5,06E+05	8,17E+05	3,38E+05	5,06E+05	1,50	0,15
113	Hypotaurine	C00519	Sulfur metabolism	110,0277	1,20	+	3,25E+05	2,23E+05	5,19E+05	7,39E+04	1,09E+04	6,54E+04	3,25E+05	6,54E+04	0,20	0,03
114	3-Sulfino-L-alanine	C00606	Sulfur metabolism	152,0014	1,21	-	1,07E+04	1,73E+04	1,70E+04	2,78E+04	3,14E+04	4,53E+04	1,70E+04	3,14E+04	1,85	0,03
115	L-Cysteate	C00506	Sulfur metabolism	170,0128	1,69	+	1,82E+04	1,51E+04	1,68E+04	2,20E+04	2,43E+04	2,64E+04	1,68E+04	2,43E+04	1,45	0,01
116	L-Methionine S-oxide	C02989	Sulfur metabolism	166,0537	1,51	+	2,61E+05	3,88E+05	2,55E+05	6,93E+05	1,10E+06	1,13E+06	2,61E+05	1,10E+06	4,21	0,01
117	5-Hydroxyindoleacetate	C05635	Indole and Tryptophan	192,0659	1,30	+	2,98E+03	3,32E+03	2,14E+03	8,64E+03	1,64E+04	9,92E+03	2,98E+03	9,92E+03	3,33	0,02
118	Indole	C00463	Indole and Tryptophan	118,0657	1,27	+	7,45E+04	6,98E+04	5,25E+04	6,55E+04	1,14E+05	6,15E+04	6,98E+04	6,55E+04	0,94	0,46
119	kynurenine	C00328	Indole and Tryptophan	209,0924	1,25	+	2,26E+03	0,00E+00	2,52E+03	8,01E+04	1,56E+05	2,22E+05	2,26E+03	1,56E+05	69,01	0,02
120	N-formyl kynurenine	C02700	Indole and Tryptophan	237,0873	1,25	+	0,00E+00	0,00E+00	0,00E+00	0,00E+00	4,12E+04	7,68E+04	0,00E+00	4,12E+04	#DIV/0!	0,15
121	Glycerol 3-phosphate	C00093	Glycerophospholipid biosynthesis	173,0213	1,27	+	1,65E+06	2,03E+06	2,64E+06	2,02E+06	1,24E+06	1,09E+06	2,03E+06	1,24E+06	0,61	0,18
122	Ethanolamine phosphate	C00346	Glycerophospholipid biosynthesis	142,0268	1,23	+	5,20E+05	9,19E+05	8,32E+05	1,57E+06	1,55E+06	2,51E+06	8,32E+05	1,57E+06	1,88	0,03
123	N-Methylethanolamine phosphate	C01210	Glycerophospholipid biosynthesis	156,0425	1,13	+	2,17E+05	2,32E+05	2,18E+05	2,59E+05	3,22E+05	2,73E+05	2,18E+05	2,73E+05	1,25	0,04
124	sn-glycero-3-Phosphoethanolamine	C01233	Glycerophospholipid biosynthesis	216,0629	1,22	+	6,19E+04	1,90E+05	1,39E+05	1,43E+05	2,07E+05	1,90E+05	1,39E+05	1,90E+05	1,37	0,30
125	CDP-choline	C00307	Glycerophospholipid biosynthesis	489,1154	1,21	+	2,96E+04	4,66E+04	1,30E+05	4,66E+04	3,18E+04	9,12E+04	4,66E+04	4,66E+04	1,00	0,75
126	L-Carnitine	C00318	Carnitine and fatty acid metabolsim	162,1128	1,22	+	1,23E+07	2,06E+07	1,29E+07	5,21E+07	1,22E+08	1,36E+08	1,29E+07	1,22E+08	9,45	0,03
127	propionyl-carnitine	HMDB00824	Carnitine and fatty acid metabolsim	218,1392	1,25	+	1,12E+06	2,11E+06	1,08E+06	9,16E+06	2,08E+07	2,28E+07	1,12E+06	2,08E+07	18,51	0,02
128	butanoyl-l-carnitine	HMDB00736	Carnitine and fatty acid metabolsim	232,1548	1,25	+	1,46E+06	7,36E+05	6,28E+05	5,29E+06	3,73E+06	3,80E+06	7,36E+05	3,80E+06	5,16	0,00
129	acyl-C4-OH	HMDB13127	Carnitine and fatty acid metabolsim	248,1498	1,27	+	1,94E+05	4,85E+05	7,85E+05	5,91E+05	1,20E+06	9,26E+05	4,85E+05	9,26E+05	1,91	0,16
130	acyl-C4-DC	HMDB13133	Carnitine and fatty acid metabolsim	262,1290	1,25	+	4,11E+04	9,22E+04	7,56E+04	4,77E+04	1,31E+05	1,36E+05	7,56E+04	1,31E+05	1,73	0,34
131	acyl-C5-OH	ac107	Carnitine and fatty acid metabolsim	262,1654	1,25	+	2,14E+05	3,63E+05	3,30E+05	2,44E+06	5,45E+06	4,32E+06	3,30E+05	4,32E+06	13,06	0,01
132	Acetyl phosphate	C00227	Other	140,9957	1,45	+	5,60E+05	5,31E+05	4,06E+05	5,75E+05	2,83E+05	2,98E+05	5,31E+05	2,98E+05	0,56	0,34
133	1-Hydroxy-2-aminoethylphosphonate	C05678	Other	142,0268	1,23	+	5,20E+05	9,19E+05	8,32E+05	1,57E+06	1,55E+06	2,51E+06	8,32E+05	1,57E+06	1,88	0,03
134	N5-Methyl-L-glutamine	C03153	Other	161,0927	1,25	+	2,52E+04	1,44E+04	3,40E+04	5,22E+04	4,54E+04	5,86E+04	2,52E+04	5,22E+04	2,07	0,02
135	2-Methyleneglutarate	C02930	Other	145,0499	1,30	+	1,09E+05	1,26E+05	1,84E+05	2,26E+05	2,00E+05	2,03E+05	1,26E+05	2,03E+05	1,61	0,04

136	4-Aminobenzoate	C00568	Other	138,0553	1,89	+	9,98E+04	1,08E+05	1,17E+05	2,01E+05	2,52E+05	2,14E+05	1,08E+05	2,14E+05	1,99	0,00
137	Leucocyanidin	C05906	Other	307,0838	1,25	+	7,23E+04	1,09E+05	9,00E+04	7,16E+04	9,10E+04	5,95E+04	9,00E+04	7,16E+04	0,80	0,31
138	beta-Butoxyethyl nicotinate	C13138	Other	224,1286	1,24	+	2,93E+05	2,59E+05	3,05E+05	2,76E+05	1,97E+05	2,48E+05	2,93E+05	2,48E+05	0,85	0,17
139	3-Oxalomalate	C01990	Other	207,0147	1,20	+	3,17E+04	6,78E+04	5,61E+04	8,70E+04	1,63E+05	2,85E+05	5,61E+04	1,63E+05	2,91	0,10
140	Diallyl sulfide	C08370	Other	115,0574	1,09	+	1,71E+03	4,67E+03	5,28E+03	2,82E+04	5,87E+04	4,78E+04	4,67E+03	4,78E+04	10,24	0,01
141	D-glucono-1,5-lactone	C00198	Other	179,0550	1,09	+	7,89E+03	2,45E+04	1,27E+04	3,83E+04	4,40E+04	6,51E+04	1,27E+04	4,40E+04	3,47	0,02
142	2-Dehydro-3-deoxy-D-glucarate	C03921	Other	193,0350	1,58	+	0,00E+00	2,43E+03	4,38E+03	7,76E+03	2,90E+04	2,90E+04	2,43E+03	2,90E+04	11,93	0,05
143	Ferric gluconate	C13511	Other	196,0581	1,10	+	1,81E+04	1,11E+04	4,34E+03	2,44E+04	3,11E+04	3,04E+04	1,11E+04	3,04E+04	2,74	0,02
144	6-Thioxanthine 5--monophosphate	C16618	Other	381,0237	1,13	+	4,35E+04	2,67E+04	0,00E+00	7,48E+04	1,03E+04	1,40E+04	2,67E+04	1,40E+04	0,53	0,71
145	Deoxyribose triphosphate	C12347	Other	374,9649	1,15	+	0,00E+00	0,00E+00	0,00E+00	0,00E+00	0,00E+00	0,00E+00	0,00E+00	0,00E+00	#DIV/0!	#DIV/0!
146	Riboflavin	C00255	Other	377,1474	1,26	+	2,96E+05	2,53E+05	1,82E+05	8,58E+04	2,22E+05	1,28E+05	2,53E+05	1,28E+05	0,51	0,13
147	N-Amidino-L-aspartate	C03139	Other	176,0662	1,25	+	1,90E+04	3,57E+04	4,41E+04	6,87E+04	2,42E+04	6,19E+03	3,57E+04	2,42E+04	0,68	1,00
148	threo-3-Hydroxy-L-aspartate	C11511	Other	150,0388	1,49	+	1,39E+04	2,69E+04	1,86E+04	4,00E+04	6,23E+04	6,55E+04	1,86E+04	6,23E+04	3,34	0,02
149	N-Carbamyl-L-glutamate	C05829	Other	191,0673	1,21	+	3,08E+04	3,45E+04	4,40E+04	1,97E+05	2,46E+05	3,67E+05	3,45E+04	2,46E+05	7,12	0,01
