# Supplementary material for: Danon Disease-Associated LAMP-2 Deficiency Drives Metabolic Signature Indicative of Mitochondrial Aging and Fibrosis in Cardiac Tissue and hiPSC-Derived Cardiomyocytes
Source: J Clin Med. 2020 Jul 31;9(8):2457. doi: 10.3390/jcm9082457 (PMC7465084; doi:10.3390/jcm9082457)
Supplement: Supplementary file 1 [file jcm-09-02457-s001.zip › Supplementary Material Del Favero MDPI_JCM/Supplementary Material 2_Metabolome Analysis MEDIUM.pdf]

hiPS-CM Metabolome analysis MEDIUM

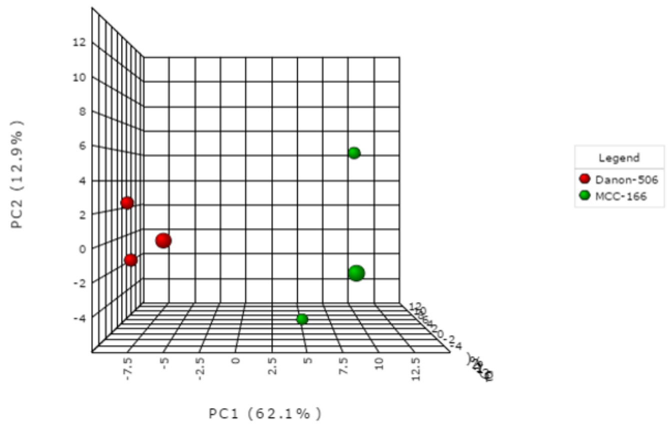

Principal Component Analysis

RAW DATA

							MCC-166_Controls			Danon-506			MEDIAN		FOLD CHANGE	p-value
N.	compound	CmpdID	Pathway	parent	med Rt	Polarity	M1	M2	M3	D1	D2	D3	M	D	Danon/MCC	M
1	L-alanine	C00041	Amino acids	90,0559	1,20	+	3,79E+07	3,86E+07	4,97E+07	4,91E+07	5,37E+07	4,84E+07	3,86E+07	4,91E+07	1,27	0,12
2	L-arginine	C00062	Amino acids	175,1194	1,18	+	1,29E+08	1,28E+08	1,17E+08	1,50E+08	1,60E+08	1,70E+08	1,28E+08	1,60E+08	1,25	0,01
3	L-asparagine	C00152	Amino acids	133,0612	1,20	+	3,87E+06	4,18E+06	4,53E+06	5,07E+06	4,16E+06	4,85E+06	4,18E+06	4,85E+06	1,16	0,21
4	L-aspartate	C00049	Amino acids	134,0452	1,22	+	4,52E+05	4,54E+05	3,78E+05	7,96E+05	8,66E+05	9,16E+05	4,52E+05	8,66E+05	1,92	0,00
5	L-cysteine	C00097	Amino acids	122,0276	1,23	+	1,32E+06	1,30E+06	1,23E+06	4,35E+06	3,80E+06	4,03E+06	1,30E+06	4,03E+06	3,10	0,00
6	L-glutamate	C00025	Amino acids	148,0608	1,21	+	7,38E+06	7,14E+06	6,55E+06	2,03E+07	2,42E+07	2,43E+07	7,14E+06	2,42E+07	3,39	0,00
7	L-glutamine	C00064	Amino acids	147,0768	1,20	+	3,68E+07	4,28E+07	5,04E+07	2,78E+07	1,74E+07	1,50E+07	4,28E+07	1,74E+07	0,41	0,01
8	glycine	C00037	Amino acids	76,0403	1,22	+	3,06E+06	2,87E+06	3,70E+06	4,81E+06	5,42E+06	5,22E+06	3,06E+06	5,22E+06	1,71	0,00
9	L-histidine	C00135	Amino acids	156,0771	1,11	+	6,25E+06	5,45E+06	5,79E+06	5,15E+06	6,25E+06	5,27E+06	5,79E+06	5,27E+06	0,91	0,55
10	L-leucine	C00123	Amino acids	132,1023	1,24	+	2,37E+08	2,00E+08	3,13E+08	1,85E+08	1,81E+08	1,78E+08	2,37E+08	1,81E+08	0,76	0,11
11	L-lysine	C00047	Amino acids	147,1131	1,10	+	2,68E+07	2,11E+07	2,38E+07	2,43E+07	2,14E+07	2,49E+07	2,38E+07	2,43E+07	1,02	0,86
12	L-methionine	C00073	Amino acids	150,0577	1,24	+	2,53E+07	2,01E+07	2,93E+07	2,24E+07	2,27E+07	2,35E+07	2,53E+07	2,27E+07	0,90	0,48

13	L-phenylalanine	C00079	Amino acids	166,0861	1,24	+	4,06E+07	3,58E+07	3,89E+07	3,65E+07	4,03E+07	3,54E+07	3,89E+07	3,65E+07	0,94	0,65
14	L-proline	C00148	Amino acids	116,0712	1,23	+	1,67E+08	1,52E+08	2,08E+08	2,27E+08	2,34E+08	2,11E+08	1,67E+08	2,27E+08	1,36	0,05
15	L-serine	C00065	Amino acids	106,0506	1,20	+	1,62E+06	1,68E+06	1,64E+06	1,33E+06	1,04E+06	1,25E+06	1,64E+06	1,25E+06	0,76	0,01
16	L-threonine	C00188	Amino acids	120,0661	1,21	+	6,06E+06	6,69E+06	8,38E+06	7,39E+06	7,02E+06	6,47E+06	6,69E+06	7,02E+06	1,05	0,92
17	L-tryptophan	C00078	Amino acids	205,0976	1,34	+	6,80E+06	9,35E+06	4,74E+06	2,80E+06	4,97E+06	4,41E+06	6,80E+06	4,41E+06	0,65	0,12
18	L-tyrosine	C00082	Amino acids	182,0815	1,24	+	2,04E+07	1,80E+07	2,43E+07	1,80E+07	2,08E+07	2,08E+07	2,04E+07	2,08E+07	1,02	0,65
19	L-valine	C00183	Amino acids	118,0869	1,24	+	5,42E+07	4,64E+07	7,07E+07	4,60E+07	4,48E+07	4,65E+07	5,42E+07	4,60E+07	0,85	0,19
20	L-cystine	C00491	Amino acids	241,0310	1,24	+	1,99E+06	2,17E+06	2,56E+06	1,59E+06	1,17E+06	1,10E+06	2,17E+06	1,17E+06	0,54	0,01
21	ADP	C00008	Nucleotides	426,0232	1,48	-	7,71E+03	2,65E+03	2,73E+03	3,41E+03	4,52E+03	1,10E+03	2,73E+03	3,41E+03	1,25	0,53
22	AMP	C00020	Nucleotides	348,0710	1,26	+	4,92E+03	0,00E+00	5,84E+03	3,93E+04	4,41E+04	2,93E+04	4,92E+03	3,93E+04	7,98	0,00
23	Adenosine	C00212	Nucleotides	268,1044	1,27	+	3,82E+03	0,00E+00	5,44E+03	4,60E+03	7,07E+03	9,44E+03	3,82E+03	7,07E+03	1,85	0,14
24	Adenine	C00147	Nucleotides	136,0622	1,24	+	1,15E+06	1,34E+06	1,49E+06	1,27E+06	1,13E+06	1,23E+06	1,34E+06	1,23E+06	0,92	0,35
25	Guanine	C00242	Nucleotides	152,0571	1,25	+	1,37E+04	0,00E+00	1,69E+04	0,00E+00	0,00E+00	8,60E+03	1,37E+04	0,00E+00	0,00	0,28
26	Cytidine	C00475	Nucleotides	244,0932	1,24	+	7,29E+04	6,73E+04	7,52E+04	2,18E+05	2,06E+05	2,58E+05	7,29E+04	2,18E+05	2,99	0,00
27	Cytosine	C00380	Nucleotides	112,0513	1,24	+	1,57E+05	1,09E+05	1,30E+05	3,95E+05	4,38E+05	7,01E+05	1,30E+05	4,38E+05	3,37	0,02
28	Thymine	C00178	Nucleotides	127,0507	1,23	+	1,41E+05	1,86E+05	1,49E+05	1,30E+05	8,49E+04	1,25E+05	1,49E+05	1,25E+05	0,83	0,08
29	UDP	C00015	Nucleotides	405,0103	1,79	+	2,15E+03	0,00E+00	5,88E+04	2,78E+04	2,16E+03	1,82E+03	2,15E+03	2,16E+03	1,00	0,67
30	Hypoxanthine	C00262	Nucleotides	137,0462	1,25	+	1,19E+06	1,22E+06	1,82E+06	1,01E+06	7,46E+05	3,01E+05	1,22E+06	7,46E+05	0,61	0,07
31	Xanthine	C00385	Nucleotides	151,0253	1,59	-	2,15E+05	2,43E+05	1,39E+05	1,63E+05	1,15E+05	9,26E+04	2,15E+05	1,15E+05	0,53	0,11
32	Allantoate	C00499	Nucleotides	177,0613	1,13	+	5,03E+06	4,33E+06	7,77E+06	2,80E+06	2,42E+06	2,14E+06	5,03E+06	2,42E+06	0,48	0,04
33	5-Hydroxyisourate	C11821	Nucleotides	185,0302	1,08	+	1,09E+03	0,00E+00	4,72E+03	3,13E+03	4,72E+03	1,16E+03	1,09E+03	3,13E+03	2,86	0,58
34	Urate	C00366	Nucleotides	167,0215	1,24	-	1,77E+05	1,98E+05	1,78E+05	1,11E+05	1,26E+05	1,15E+05	1,78E+05	1,15E+05	0,65	0,00
35	5-6-Dihydrothymine	C00906	Nucleotides	129,0664	1,23	+	1,40E+06	1,21E+06	1,53E+06	1,50E+06	1,55E+06	1,54E+06	1,40E+06	1,54E+06	1,10	0,20
36	Pyridoxal	C00250	Nucleotides	168,0659	1,24	+	2,09E+06	2,05E+06	2,26E+06	1,56E+06	1,37E+06	1,61E+06	2,09E+06	1,56E+06	0,75	0,00
37	4-Pyridoxate	C00847	Nucleotides	184,0601	1,26	+	3,40E+04	1,20E+04	1,79E+04	9,47E+03	2,37E+05	1,67E+05	1,79E+04	1,67E+05	9,32	0,16
38	Pyridoxamine 5'-phosphate	C00647	Nucleotides	249,0624	1,24	+	8,88E+05	1,12E+06	5,17E+05	2,79E+05	4,47E+05	4,65E+05	8,88E+05	4,47E+05	0,50	0,07
39	Nicotinamide	C00153	Nucleotides	123,0558	1,25	+	5,72E+06	5,07E+06	5,42E+06	3,81E+05	3,61E+05	3,94E+05	5,42E+06	3,81E+05	0,07	0,00

40	Nicotinate ribonucleotide	C01185	Nucleotides	335,0457	1,13	-	4,15E+04	3,97E+04	7,39E+04	2,49E+04	2,16E+04	2,71E+04	4,15E+04	2,49E+04	0,60	0,07
41	Phosphate	C00009	Phosphates	98,9850	1,25	+	6,03E+07	6,46E+07	6,90E+07	6,46E+07	5,97E+07	5,64E+07	6,46E+07	5,97E+07	0,92	0,27
42	Diphosphate	C00013	Phosphates	176,9353	1,27	-	2,95E+06	3,45E+06	3,04E+06	3,08E+06	2,68E+06	3,09E+06	3,04E+06	3,08E+06	1,01	0,39
43	D-Glucose	C00031	Glycolysis	179,0559	1,18	-	8,12E+04	5,19E+04	4,13E+04	2,31E+05	1,91E+05	3,12E+05	5,19E+04	2,31E+05	4,46	0,01
44	D-Glucose 6-phosphate	C02965	Glycolysis	259,0228	1,27	-	4,48E+04	3,01E+04	2,04E+04	4,81E+04	2,70E+04	4,06E+04	3,01E+04	4,06E+04	1,35	0,51
45	D-Fructose 1-6-bisphosphate	C00354	Glycolysis	338,9892	1,64	-	4,27E+03	4,03E+03	1,41E+04	2,86E+04	1,19E+04	7,26E+03	4,27E+03	1,19E+04	2,79	0,31
46	D-Glyceraldehyde 3-phosphate/Glycerone phosphate	C00118	Glycolysis	168,9891	1,27	-	5,05E+04	4,58E+04	5,90E+04	7,07E+04	5,75E+04	8,66E+04	5,05E+04	7,07E+04	1,40	0,10
47	2/3-Phospho-D-glycerate	C00631	Glycolysis	184,9851	1,31	-	6,49E+04	7,66E+04	1,11E+05	8,78E+04	7,54E+04	1,06E+05	7,66E+04	8,78E+04	1,15	0,76
48	Phosphoenolpyruvate	C00074	Glycolysis	166,9744	1,40	-	5,50E+03	3,58E+03	6,40E+03	7,57E+03	3,15E+03	7,67E+03	5,50E+03	7,57E+03	1,38	0,60
49	Pyruvate	C00022	Glycolysis	87,0074	1,36	-	4,69E+06	4,58E+06	5,41E+06	4,99E+06	5,82E+06	5,68E+06	4,69E+06	5,68E+06	1,21	0,18
50	Lactate	C01432	Glycolysis	89,0230	1,28	-	1,69E+08	1,76E+08	1,91E+08	1,20E+08	1,27E+08	1,08E+08	1,76E+08	1,20E+08	0,68	0,00
51	Maltose	C00208	Other sugars	343,1236	1,17	+	1,95E+04	1,65E+04	8,01E+03	0,00E+00	1,91E+04	3,28E+04	1,65E+04	1,91E+04	1,16	0,81
52	Citrate	C00158	TCA cycle	191,0194	1,26	-	6,13E+06	7,04E+06	9,12E+06	2,10E+07	2,15E+07	2,18E+07	7,04E+06	2,15E+07	3,05	0,00
53	2-Oxoglutarate	C00026	TCA cycle	145,0143	1,28	-	5,25E+05	5,41E+05	5,10E+05	1,32E+06	1,36E+06	1,34E+06	5,25E+05	1,34E+06	2,54	0,00
54	Succinate	C00042	TCA cycle	117,0181	1,29	-	4,91E+05	4,22E+05	7,41E+05	4,65E+05	3,79E+05	4,11E+05	4,91E+05	4,11E+05	0,84	0,25
55	Fumarate	C00122	TCA cycle	115,0025	1,29	-	2,17E+05	2,16E+05	3,20E+05	3,43E+05	3,56E+05	4,08E+05	2,17E+05	3,56E+05	1,64	0,04
56	Malate	C00149	TCA cycle	133,0131	1,28	-	1,64E+06	1,82E+06	2,65E+06	3,28E+06	3,91E+06	3,59E+06	1,82E+06	3,59E+06	1,97	0,01
57	2-Hydroxyglutarate/Citramalate	C02630	Alternative Carboxylic acids	147,0291	1,25	-	1,83E+05	2,00E+05	2,68E+05	5,67E+05	5,89E+05	4,54E+05	2,00E+05	5,67E+05	2,83	0,00
58	Sedoheptulose 7-phosphate	C05382	Pentose Phosphate Pathway	289,0346	1,28	-	3,80E+03	7,99E+03	3,69E+03	2,00E+04	1,00E+04	2,30E+04	3,80E+03	2,00E+04	5,26	0,04
59	Pentose phosphates (isobars)	C00199	Pentose Phosphate Pathway	229,0117	1,26	-	4,78E+04	9,02E+04	1,04E+05	9,21E+04	5,94E+04	6,34E+04	9,02E+04	6,34E+04	0,70	0,68
60	Glutathione	C00051	GSH homeostasis	308,0916	1,25	+	1,21E+04	0,00E+00	0,00E+00	0,00E+00	0,00E+00	0,00E+00	0,00E+00	0,00E+00	#DIV/0!	0,37
61	5-Oxoproline	C01879	GSH homeostasis	130,0503	1,23	+	1,01E+08	9,58E+07	9,91E+07	8,11E+07	7,76E+07	7,31E+07	9,91E+07	7,76E+07	0,78	0,00
62	S-Glutathionyl-L-cysteine	C05526	GSH homeostasis	427,0963	1,23	+	7,46E+03	0,00E+00	3,86E+03	6,43E+03	0,00E+00	0,00E+00	3,86E+03	0,00E+00	0,00	0,62
63	Cys-Gly	C01419	GSH homeostasis	179,0489	1,24	+	1,48E+04	0,00E+00	0,00E+00	0,00E+00	0,00E+00	0,00E+00	0,00E+00	0,00E+00	#DIV/0!	0,37
64	Ascorbate	C00072	GSH homeostasis	175,0261	1,23	-	4,42E+04	4,59E+04	3,37E+04	7,38E+04	9,26E+04	9,37E+04	4,42E+04	9,26E+04	2,09	0,00
65	Dehydroascorbate	C05422	GSH homeostasis	173,0088	1,25	-	6,90E+05	7,65E+05	9,89E+05	1,84E+06	1,98E+06	1,74E+06	7,65E+05	1,84E+06	2,40	0,00
66	gamma-L-Glutamyl-D-alanine	C03738	Gamma-glutamyls	219,0983	1,25	+	1,36E+04	4,29E+04	1,64E+04	1,50E+04	3,70E+04	1,84E+04	1,64E+04	1,84E+04	1,12	0,94
67	gamma-Glutamyl-gamma-aminobutyrate	C15767	Gamma-glutamyls	233,1143	1,25	+	5,00E+04	3,92E+04	2,37E+04	6,68E+04	2,41E+04	3,14E+04	3,92E+04	3,14E+04	0,80	0,85

68	gamma-L-Glutamylputrescine	C15699	Gamma-glutamyls	218,1506	1,09	+	1,20E+03	0,00E+00	1,19E+03	0,00E+00	0,00E+00	0,00E+00	1,19E+03	0,00E+00	0,00	0,12
69	(5-L-Glutamyl)-L-glutamine	C05283	Gamma-glutamyls	276,1196	1,23	+	1,18E+05	1,51E+05	1,25E+05	6,05E+04	7,78E+04	3,03E+04	1,25E+05	6,05E+04	0,48	0,01
70	Dimethylglycine	C01026	Serine biosynthesis and one-carbon metabolism	104,0714	1,29	+	1,32E+06	5,74E+05	4,24E+05	2,99E+05	3,12E+05	3,27E+05	5,74E+05	3,12E+05	0,54	0,17
71	3-Phosphonopyruvate	C02798	Serine biosynthesis and one-carbon metabolism	168,9901	1,40	+	3,72E+03	2,65E+03	5,79E+03	1,58E+04	1,02E+04	9,76E+03	3,72E+03	1,02E+04	2,73	0,02
72	Ornithine	C01602	Urea cycle	133,0976	1,10	+	1,63E+07	1,38E+07	2,07E+07	8,94E+06	6,67E+06	7,16E+06	1,63E+07	7,16E+06	0,44	0,01
73	L-Citrulline	C00327	Urea cycle	176,1035	1,21	+	1,57E+04	0,00E+00	0,00E+00	0,00E+00	0,00E+00	1,92E+04	0,00E+00	0,00E+00	#DIV/0!	0,89
74	Putrescine	C00134	Polyamines	89,1081	1,15	+	1,48E+07	1,45E+07	2,23E+07	1,87E+07	1,33E+07	1,01E+07	1,48E+07	1,33E+07	0,90	0,43
75	Spermidine	C00315	Polyamines	146,1655	1,13	+	6,64E+05	4,86E+05	5,99E+05	1,71E+06	2,32E+06	2,29E+06	5,99E+05	2,29E+06	3,83	0,00
76	Spermine	C00750	Polyamines	203,2234	1,15	+	5,00E+04	2,67E+04	5,02E+04	7,73E+04	4,72E+04	4,75E+04	5,00E+04	4,75E+04	0,95	0,30
77	N-Acetylneuraminate	C00270	Aminosugars	310,1138	1,21	+	3,58E+03	0,00E+00	2,94E+03	1,52E+03	2,66E+03	4,09E+03	2,94E+03	2,66E+03	0,91	0,68
78	5-Guanidino-2-oxopentanoate	C03771	Arginine and proline metabolism	174,0879	1,24	+	7,69E+04	5,24E+04	9,54E+04	1,16E+05	1,68E+05	1,66E+05	7,69E+04	1,66E+05	2,16	0,02
79	Phosphocreatine	C02305	Arginine and proline metabolism	212,0434	1,27	+	5,86E+03	1,47E+04	4,94E+03	4,04E+03	4,61E+03	0,00E+00	5,86E+03	4,04E+03	0,69	0,18
80	Creatine	C00300	Arginine and proline metabolism	132,0772	1,23	+	4,16E+06	2,73E+06	2,92E+06	7,62E+05	7,48E+05	5,53E+05	2,92E+06	7,48E+05	0,26	0,00
81	Creatinine	C00791	Arginine and proline metabolism	114,0668	1,22	+	2,08E+05	1,92E+05	2,88E+05	3,99E+04	5,49E+04	3,84E+04	2,08E+05	3,99E+04	0,19	0,00
82	Guanidinoacetate	C00581	Arginine and proline metabolism	118,0617	1,22	+	2,19E+04	1,29E+04	5,97E+04	1,04E+05	4,76E+05	4,72E+05	2,19E+04	4,72E+05	21,54	0,06
83	trans-4-Hydroxy-L-proline	C01157	Arginine and proline metabolism	132,0660	1,21	+	1,56E+07	1,89E+07	2,13E+07	2,11E+07	2,01E+07	1,94E+07	1,89E+07	2,01E+07	1,06	0,41
84	5-Aminopentanoate	C00431	Arginine and proline metabolism	118,0869	1,64	+	3,03E+06	2,82E+06	2,86E+06	2,85E+06	2,88E+06	0,00E+00	2,86E+06	2,85E+06	1,00	0,36
85	L-1-Pyrroline-3-hydroxy-5-carboxylate	C04281	Arginine and proline metabolism	130,0503	1,80	+	2,50E+07	2,11E+07	2,21E+07	2,02E+07	1,99E+07	1,93E+07	2,21E+07	1,99E+07	0,90	0,07
86	Pantothenol	C00864	Panthothenate metabolism	220,1184	1,33	+	6,00E+05	5,43E+05	5,88E+05	5,39E+05	5,98E+05	5,34E+05	5,88E+05	5,39E+05	0,92	0,50
87	Pantetheine	C00831	Panthothenate metabolism	279,1355	1,26	+	1,05E+04	2,72E+03	0,00E+00	0,00E+00	0,00E+00	0,00E+00	2,72E+03	0,00E+00	0,00	0,23
88	Taurine	C00245	Sulfur metabolism	126,0224	1,25	+	5,20E+03	2,09E+03	0,00E+00	0,00E+00	0,00E+00	0,00E+00	2,09E+03	0,00E+00	0,00	0,18
89	Hypotaurine	C00519	Sulfur metabolism	110,0277	1,20	+	3,92E+04	1,63E+04	1,28E+04	0,00E+00	0,00E+00	0,00E+00	1,63E+04	0,00E+00	0,00	0,05
90	3-Sulfino-L-alanine	C00606	Sulfur metabolism	152,0014	1,21	-	1,02E+04	9,71E+03	1,14E+04	1,10E+04	4,40E+03	5,99E+03	1,02E+04	5,99E+03	0,59	0,18
91	L-Cysteate	C00506	Sulfur metabolism	170,0128	1,69	+	1,18E+05	1,28E+05	1,42E+05	1,15E+05	9,55E+04	1,07E+05	1,28E+05	1,07E+05	0,84	0,06
92	L-Methionine S-oxide	C02989	Sulfur metabolism	166,0537	1,51	+	1,01E+07	8,16E+06	9,34E+06	8,37E+06	8,54E+06	9,05E+06	9,34E+06	8,54E+06	0,91	0,41
93	Indole	C00463	Indole and Tryptophan	118,0657	1,27	+	4,41E+05	5,10E+05	4,39E+05	4,31E+05	4,76E+05	4,70E+05	4,41E+05	4,70E+05	1,06	0,88
94	kynurenine	C00328	Indole and Tryptophan	209,0924	1,25	+	1,46E+04	0,00E+00	1,07E+04	1,48E+06	1,49E+06	1,43E+06	1,07E+04	1,48E+06	138,36	0,00
95	N-formyl kynurenine	C02700	Indole and Tryptophan	237,0873	1,25	+	1,23E+04	4,49E+03	0,00E+00	1,29E+05	8,46E+04	1,18E+05	4,49E+03	1,18E+05	26,26	0,00

96	Glycerol 3-phosphate	C00093	Glycerophospholipid biosynthesis	173,0213	1,27	+	4,31E+04	3,09E+04	3,74E+04	3,58E+04	2,47E+04	2,48E+04	3,74E+04	2,48E+04	0,66	0,16
97	Ethanolamine phosphate	C00346	Glycerophospholipid biosynthesis	142,0268	1,23	+	5,27E+04	7,44E+04	5,03E+04	1,18E+05	1,21E+05	1,07E+05	5,27E+04	1,18E+05	2,25	0,00
98	N-Methylethanolamine phosphate	C01210	Glycerophospholipid biosynthesis	156,0425	1,13	+	8,22E+04	6,48E+04	5,91E+04	6,89E+04	7,05E+04	4,92E+04	6,48E+04	6,89E+04	1,06	0,58
99	sn-glycero-3-Phosphoethanolamine	C01233	Glycerophospholipid biosynthesis	216,0629	1,22	+	2,21E+04	8,10E+03	0,00E+00	0,00E+00	3,10E+04	7,33E+04	8,10E+03	3,10E+04	3,83	0,33
100	L-Carnitine	C00318	Carnitine and fatty acid metabolsim	162,1128	1,22	+	5,65E+07	5,20E+07	6,38E+07	5,09E+07	6,29E+07	7,23E+07	5,65E+07	6,29E+07	1,11	0,55
101	propionyl-carnitine	HMDB00824	Carnitine and fatty acid metabolsim	218,1392	1,25	+	1,40E+06	1,12E+06	1,09E+06	4,67E+06	7,47E+06	8,78E+06	1,12E+06	7,47E+06	6,66	0,01
102	butanoyl-l-carnitine	HMDB00736	Carnitine and fatty acid metabolsim	232,1548	1,25	+	6,63E+05	5,09E+05	4,16E+05	1,47E+06	1,01E+06	1,04E+06	5,09E+05	1,04E+06	2,04	0,02
103	acyl-C4-OH	HMDB13127	Carnitine and fatty acid metabolsim	248,1498	1,27	+	5,98E+03	3,49E+04	1,19E+04	3,37E+04	3,74E+04	2,90E+04	1,19E+04	3,37E+04	2,84	0,16
104	acyl-C5-OH	ac107	Carnitine and fatty acid metabolsim	262,1654	1,25	+	1,10E+05	9,75E+04	1,38E+05	2,45E+05	3,10E+05	3,92E+05	1,10E+05	3,10E+05	2,83	0,01
105	Acetyl phosphate	C00227	Other	140,9957	1,45	+	4,72E+04	7,52E+04	7,62E+04	7,69E+04	6,36E+04	7,39E+04	7,52E+04	7,39E+04	0,98	0,64
106	1-Hydroxy-2-aminoethylphosphonate	C05678	Other	142,0268	1,23	+	5,27E+04	7,44E+04	5,03E+04	1,18E+05	1,21E+05	1,07E+05	5,27E+04	1,18E+05	2,25	0,00
107	2-Methyleneglutarate	C02930	Other	145,0499	1,30	+	2,70E+04	2,88E+04	2,15E+04	7,29E+04	7,42E+04	9,51E+04	2,70E+04	7,42E+04	2,74	0,00
108	4-Aminobenzoate	C00568	Other	138,0553	1,89	+	9,97E+04	8,45E+04	1,06E+05	9,90E+04	8,80E+04	9,33E+04	9,97E+04	9,33E+04	0,94	0,66
109	beta-Butoxyethyl nicotinate	C13138	Other	224,1286	1,24	+	8,10E+04	1,14E+05	1,27E+05	1,31E+05	1,42E+05	1,68E+05	1,14E+05	1,42E+05	1,24	0,09
110	3-Oxalomalate	C01990	Other	207,0147	1,20	+	1,57E+06	1,53E+06	1,69E+06	8,00E+05	5,31E+05	4,88E+05	1,57E+06	5,31E+05	0,34	0,00
111	D-glucono-1,5-lactone	C00198	Other	179,0550	1,09	+	8,95E+03	3,43E+03	3,28E+03	6,85E+03	6,90E+03	1,24E+04	3,43E+03	6,90E+03	2,01	0,25
112	2-Dehydro-3-deoxy-D-glucarate	C03921	Other	193,0350	1,58	+	1,01E+05	6,81E+04	7,66E+04	2,23E+05	0,00E+00	0,00E+00	7,66E+04	0,00E+00	0,00	0,93
113	Ferric gluconate	C13511	Other	196,0581	1,10	+	0,00E+00	0,00E+00	4,06E+03	1,81E+03	3,01E+03	4,52E+03	0,00E+00	3,01E+03	#DIV/0!	0,32
114	6-Thioxanthine 5--monophosphate	C16618	Other	381,0237	1,13	+	9,49E+05	9,12E+05	1,18E+06	4,14E+05	4,91E+05	3,93E+05	9,49E+05	4,14E+05	0,44	0,00
115	Deoxyribose triphosphate	C12347	Other	374,9649	1,15	+	5,09E+05	5,15E+05	5,85E+05	2,93E+05	2,68E+05	2,64E+05	5,15E+05	2,68E+05	0,52	0,00
116	Riboflavin	C00255	Other	377,1474	1,26	+	3,69E+05	4,50E+05	5,50E+05	6,35E+05	5,22E+05	4,44E+05	4,50E+05	5,22E+05	1,16	0,37
117	N-Amidino-L-aspartate	C03139	Other	176,0662	1,25	+	5,30E+06	4,73E+06	5,70E+06	3,58E+06	3,33E+06	3,00E+06	5,30E+06	3,33E+06	0,63	0,00
118	threo-3-Hydroxy-L-aspartate	C11511	Other	150,0388	1,49	+	5,94E+05	5,82E+05	6,16E+05	6,28E+05	6,40E+05	6,28E+05	5,94E+05	6,28E+05	1,06	0,03
119	N-Carbamyl-L-glutamate	C05829	Other	191,0673	1,21	+	1,27E+04	3,71E+03	1,54E+03	1,37E+04	7,51E+03	2,21E+04	3,71E+03	1,37E+04	3,70	0,19
