# Supplementary material for: Danon Disease-Associated LAMP-2 Deficiency Drives Metabolic Signature Indicative of Mitochondrial Aging and Fibrosis in Cardiac Tissue and hiPSC-Derived Cardiomyocytes
Source: J Clin Med. 2020 Jul 31;9(8):2457. doi: 10.3390/jcm9082457 (PMC7465084; doi:10.3390/jcm9082457)
Supplement: Supplementary file 1 [file jcm-09-02457-s001.zip › Supplementary Material Del Favero MDPI_JCM/Supplementary Material 3_Transcriptome Analysis TISSUE.pdf]

Tissue Transcriptome analysis FDR <0.05

>10	10-3	3-2	2-1	1-2	2-3	3-10	>10
DECREASE				INCREASE			

Common_name	Control_mean	Danon_mean	Fold_change	P-value
LAMP2	30,72	0,85	-36,14	7,28E-06
F5	5,94	0,31	-19,16	0,00105
CORIN	23,38	1,97	-11,87	0,00025064
SMTNL2	11,6	1,74	-6,67	7,16E-05
GRB14	11,83	2,23	-5,3	0,00098974
RP11-6F2.6	87,68	23,89	-3,67	0,00103745
GPR133	21,23	6,39	-3,32	0,00028001
PPIL1	52,05	19,81	-2,63	5,74E-07
ADCK3	150,46	59,69	-2,52	0,00029619
C15orf59	35,36	14,88	-2,38	0,00037795
WFS1	38,2	17,12	-2,23	0,00021408
RHOD	38,24	17,38	-2,2	0,00045086
PRDX6	244,51	117,5	-2,08	0,00048693
TEAD2	33,33	18,04	-1,85	0,00010564
SDCBP	94,19	55,09	-1,71	0,000489
CHPT1	87,92	53,52	-1,64	0,00062752
RCAN2	104,92	64,19	-1,63	0,00053559
KLC2	15,33	9,5	-1,61	0,00032412
PPPDE2	18,61	12,28	-1,52	3,44E-05
FBLIM1	31,76	21,35	-1,49	0,00097569
PMF1	54,8	42,95	-1,28	0,00042346
H2AFV	74,66	92,51	1,24	0,0003713
MRFAP1L1	32,89	43,2	1,31	0,00027617
KIAA1191	31,35	41,4	1,32	0,00091232
SCO1	16,75	22,46	1,34	0,00018585
VPS24	50,62	68,38	1,35	0,0007452
GATA6	18,52	26,01	1,4	0,00074194
TMEM106C	25,52	36,05	1,41	0,00031849
BCL2L2	17,28	25	1,45	0,00093997
MEAF6	20,23	29,7	1,47	0,00064068
RAB12	34,88	53,73	1,54	0,00103041
AC005840.1	11,78	19,62	1,67	0,00012758
LETMD1	12,52	22,34	1,78	0,00062263
PRMT2	30,3	54,9	1,81	0,00041614
WEE1	16,7	33,41	2	0,0003435
DDIT3	18,17	37,23	2,05	0,00065846
SOCS2	9,75	20,25	2,08	0,00017838
LYPLAL1	28,56	62,07	2,17	0,00039265
CHCHD6	5,77	13,01	2,25	7,02E-05
KCNJ4	10,97	24,83	2,26	0,00077827

ARHGAP24	5,4	12,48	2,31	0,00072325
C14orf135	6,75	16,32	2,42	0,00055355
IFI44L	4,28	10,81	2,53	0,0004469
JAK2	3,79	10,42	2,75	0,00069494
GATM	6,82	20,5	3,01	0,00105578
FEZ1	20,66	64,54	3,12	0,00049485
MAL	2,57	8,07	3,14	0,00080498
GADD45A	23,72	75,7	3,19	0,00084384
FAM69C	3,31	10,6	3,2	0,00013241
GPNMB	82,66	307,47	3,72	0,00010673
IDI2-AS1	6,71	27,8	4,14	0,00016254
GADD45G	21,95	100,32	4,57	0,00098244
HCN2	4,71	22,61	4,8	4,31E-05
CCL5	1,38	8,89	6,44	0,00054022
CENPA	0,62	9,1	14,68	3,98E-05
BIRC7	0,18	7,57	42,06	1,22E-05
