# Supplementary material for: Danon Disease-Associated LAMP-2 Deficiency Drives Metabolic Signature Indicative of Mitochondrial Aging and Fibrosis in Cardiac Tissue and hiPSC-Derived Cardiomyocytes
Source: J Clin Med. 2020 Jul 31;9(8):2457. doi: 10.3390/jcm9082457 (PMC7465084; doi:10.3390/jcm9082457)
Supplement: Supplementary file 1 [file jcm-09-02457-s001.zip › Supplementary Material Del Favero MDPI_JCM/Supplementary Material 4_Transcriptome Analysis CELLS.pdf]

hiPS-CM Transcriptome analysis FDR <0.01

>10	10-3	3-2	2-1	1-2	2-3	3-10	>10
DECREASE				INCREASE			

Common_name	Control_mean	Danon_mean	Fold_change	Pvalue
RP11-11N5.3	6,79	0,08	-84,88	1,09E-05
RPS4XP22	81,67	1,14	-71,64	1,20E-06
RP11-11N5.1	8,02	0,15	-53,47	0,00014414
FAR2P1	15,73	0,71	-22,15	0,0003632
PRG4	103,77	4,69	-22,13	0,00013289
LAMP2	138,15	7,44	-18,57	3,24E-09
LRFN5	10,31	0,57	-18,09	0,00030552
JPH4	22,08	1,5	-14,72	0,00036037
RARRES2	231,23	16,34	-14,15	0,00030567
**COL1A2**	**4622,43**	**361,46**	-12,79	0,00035762
SMOC2	94,93	7,58	-12,52	0,00031974
INHBE	6,39	0,57	-11,21	4,53E-06
ALDH1L2	21,67	2,14	-10,13	5,05E-05
NTNG1	12,94	1,28	-10,11	0,00023748
KCNE4	14,85	1,52	-9,77	1,78E-05
PIEZO2	68,93	7,21	-9,56	0,00037826
RP5-855D21.1	9,19	0,97	-9,47	1,15E-05
BRINP2	8,16	0,87	-9,38	1,55E-05
CD248	63,05	7,1	-8,88	0,00040496
**COL11A1**	**168,43**	**20,83**	-8,09	6,98E-05
PLAC9	74,8	9,28	-8,06	0,00027539
TMEM200A	14,69	1,86	-7,9	2,97E-05
NPR1	23,41	3,16	-7,41	0,00014172
ADAMTS5	9,2	1,25	-7,36	0,00042573
NRK	8,4	1,17	-7,18	0,00012321
GRIK2	8,53	1,19	-7,17	0,00027427
HPCAL4	7,08	0,99	-7,15	0,00018715
RP11-87H9.2	7,45	1,07	-6,96	8,44E-07
AC073610.5	34,49	5,06	-6,82	0,00042922
FAP	111,54	16,55	-6,74	0,00028863
HAS1	8,08	1,24	-6,52	0,00014815
HS6ST2	8,31	1,31	-6,34	1,04E-05
CLDN15	34,56	5,58	-6,19	1,76E-05
LINC01133	35,25	5,79	-6,09	0,00022259
ZNF248	14,75	2,43	-6,07	1,28E-05
CDON	117,9	19,45	-6,06	0,00030544
RNU1-122P	5,78	0	-6	5,59E-08
LPAR1	36,95	6,21	-5,95	9,30E-05

CBLN3	7,44	1,28	-5,81	4,50E-05
EPHB6	50,42	8,69	-5,8	0,00042459
SPON2	32,08	5,56	-5,77	0,00041355
ADGRA2	80,84	14,04	-5,76	0,0003192
DDR2	43,78	7,67	-5,71	0,00026401
GABRA2	8,78	1,59	-5,52	0,0001664
IQCA1	10,38	1,91	-5,43	0,00040822
MESTP3	33,56	6,19	-5,42	0,00013279
MESTP2	6,68	1,25	-5,34	5,75E-05
ADM2	10,61	2,04	-5,2	5,05E-07
TNXB	17,91	3,5	-5,12	0,0002142
C10orf107	9,46	1,9	-4,98	0,00028617
FKBP7	83,85	17,1	-4,9	0,00020777
MEST	542,06	110,81	-4,89	2,91E-05
RP11-736K20.5	17,98	3,7	-4,86	7,73E-05
LXN	159,37	33,67	-4,73	0,00025446
MGP	2139,43	459,89	-4,65	5,41E-06
DPP10	7,89	1,73	-4,56	0,00016096
SPARC	15044,33	3371,07	-4,46	0,00025986
MESTP1	25,19	5,67	-4,44	0,00018383
CPZ	55,87	12,86	-4,34	0,00018329
C2	10,56	2,46	-4,29	0,00035194
ASPN	31,55	7,73	-4,08	0,00040288
PSAT1	153,47	37,96	-4,04	0,00013694
SLC7A4	11,92	3,01	-3,96	0,00029075
MTND4P24	104,43	26,71	-3,91	1,01E-05
SNCAIP	53	13,6	-3,9	2,38E-05
TRAF5	36,6	9,41	-3,89	0,00030853
SERPING1	535,07	138,67	-3,86	0,00028898
LTBP2	89,46	23,3	-3,84	5,02E-05
BTBD19	20,64	5,37	-3,84	0,00015109
SULF2	282,73	74,62	-3,79	0,00010153
CLEC2D	13,74	3,66	-3,75	4,79E-05
GXYLT2	33,95	9,12	-3,72	1,12E-05
FKBP11	99,38	26,82	-3,71	0,00011418
LRP1	264,21	75,35	-3,51	7,79E-05
PKNOX2	11,25	3,27	-3,44	0,0001208
HNMT	27,98	8,13	-3,44	0,0002987
IL18R1	9,46	2,76	-3,43	0,00041644
FBXO4	9,67	2,97	-3,26	3,13E-05
PTK7	367,06	114,16	-3,22	0,00015795
IL11	7,83	2,47	-3,17	0,00011788
WISP1	12,01	3,84	-3,13	0,00020543
MAF	39,44	12,66	-3,12	0,0003673

PHGDH	299,02	96,37	-3,1	7,06E-06
TBC1D8B	8,58	2,77	-3,1	0,00034464
CCDC122	16,68	5,41	-3,08	0,00027549
PTPRG	19,18	6,27	-3,06	0,00025511
LY6E	331,69	108,96	-3,04	0,00034328
APBB2	52,08	17,81	-2,92	0,00012544
PCK2	72,25	24,87	-2,91	0,00033612
MARVELD1	131,61	45,31	-2,9	0,0003037
DNM1	63,74	22,03	-2,89	0,00027622
ZMYM6NB	41,82	14,57	-2,87	0,00017679
PXK	43,05	15,32	-2,81	2,29E-05
NEO1	159,19	57,19	-2,78	0,00032437
KIZ	57,38	20,87	-2,75	0,00022039
GCNT1	11,38	4,23	-2,69	0,00032926
KLHL29	10,85	4,07	-2,67	0,00024342
ENOX1	12,37	4,69	-2,64	9,37E-06
P4HA1	163,95	62	-2,64	0,00012944
DSE	44,94	17,44	-2,58	0,00033504
SPRY1	94,45	37,52	-2,52	0,00012959
GPX8	259,26	103,71	-2,5	0,00011293
MMP14	343,81	139,71	-2,46	6,38E-05
TPBG	31,78	13,1	-2,43	0,00016889
WARS	448,18	184,91	-2,42	0,00016246
RRBP1	174,26	72,42	-2,41	8,66E-05
ASS1	462,45	193,3	-2,39	2,26E-05
ARHGEF2	172,9	72,26	-2,39	0,00011304
SERPINH1	1147,28	484,03	-2,37	0,00027749
RNF24	25,78	10,97	-2,35	0,00042121
CEBPB	54,41	23,78	-2,29	0,000319
P4HA2	300,29	131,57	-2,28	4,72E-06
IGLV5-52	16,33	7,24	-2,26	0,00010522
C17orf51	10,34	4,57	-2,26	0,0004095
MPZL1	194,88	87,2	-2,23	0,00028591
MTHFD2P7	19,1	8,68	-2,2	2,42E-05
SLC1A5	223,91	101,77	-2,2	4,27E-05
PDIA5	61,21	27,81	-2,2	0,00025048
SNX30	15,1	6,9	-2,19	0,00023031
EPHB2	52,03	24,11	-2,16	0,0002808
C14orf132	10,73	5,06	-2,12	2,21E-05
SPG20	114,61	54,59	-2,1	0,00035983
LMF1	26,67	12,81	-2,08	0,00025736
IRAK4	11,27	5,6	-2,01	0,00012496
15.Sep	3726,12	1850,98	-2,01	0,0002318
MTHFD2	156,2	79,02	-1,98	2,40E-05

ITPR1	23,07	11,64	-1,98	0,00010864
BX322557.10	14,45	7,31	-1,98	0,0003372
NUPR1	322,12	162,93	-1,98	0,0003927
FTLP2	42,98	22,38	-1,92	0,00010986
AC011737.2	48,72	25,47	-1,91	0,00016377
SUMF2	151,96	80,25	-1,89	0,00017475
IFT20	98,34	52,11	-1,89	0,00040311
CDR2	40,83	21,72	-1,88	0,00019639
CLN5	28,8	15,64	-1,84	0,00023377
SSR2	652,19	356,99	-1,83	0,00020276
GARS	399,12	220,25	-1,81	9,41E-05
UGDH	66,4	37,58	-1,77	0,00016582
IFNAR2	33,65	18,98	-1,77	0,00020236
CDC40	30,87	17,9	-1,72	0,00011203
TMBIM4	46,6	27,12	-1,72	0,00035835
SEC61B	428,77	252,3	-1,7	0,00032599
SLC48A1	31,25	18,58	-1,68	0,00033094
CKAP4	241,24	144,19	-1,67	0,00033603
POFUT1	64,8	39,14	-1,66	0,0003982
ZFP36L1	256,4	156,56	-1,64	5,62E-05
NUDT2	44,84	27,71	-1,62	2,95E-06
MEIS3	92,77	57,29	-1,62	8,05E-05
SLC35E2B	22,59	14,31	-1,58	0,00034745
MAP2K5	26,13	16,66	-1,57	6,91E-05
SEC61A1	436,57	281,97	-1,55	3,64E-05
PLD3	1299,78	838,34	-1,55	0,00032567
SLC37A3	96,93	65,69	-1,48	1,66E-05
PTBP1	386,02	260,38	-1,48	6,91E-05
HDLBP	1727,61	1168,92	-1,48	9,72E-05
TXNDC15	161,41	109,42	-1,48	0,0001665
CNN2	362,19	249,44	-1,45	2,70E-05
YIPF5	105,44	73,56	-1,43	0,00010998
MYDGF	177,59	123,87	-1,43	0,00013164
MAN1B1	100,39	70,31	-1,43	0,00026468
ARPC5	250,77	175,84	-1,43	0,00032852
EIF2S3	209,84	148,14	-1,42	0,00015818
GLG1	122,14	87,18	-1,4	1,40E-05
SSR4	524,53	373,97	-1,4	4,34E-05
FAF2	41,34	30,04	-1,38	0,00014165
ARCN1	197,4	144,81	-1,36	0,00024514
SRPRB	97,37	73,21	-1,33	9,44E-05
SLC39A9	44,03	33,8	-1,3	0,00013247
ALDH9A1	126,52	98,74	-1,28	0,00034536
COG1	41,88	52,44	1,25	6,41E-05

SNAP29	37,31	47,26	1,27	3,81E-05
C1QBP	229,22	293,96	1,28	5,94E-05
SOD1	286,19	366,83	1,28	0,00039956
PITRM1	68,67	89,3	1,3	1,57E-05
ACOT9	67,8	89,05	1,31	1,91E-05
TSG101	203,15	267,48	1,32	6,36E-05
DCTN2	417,02	548,58	1,32	0,00024836
EIF2B2	64,42	86,18	1,34	0,00014177
EIF2B3	29,42	39,58	1,35	0,00020304
RP11-286N22.8	41,93	58,2	1,39	0,00032639
AGK	28,71	40,15	1,4	1,59E-06
ISCA2	63,97	89,36	1,4	1,75E-05
HADHA	161,51	226,52	1,4	0,00016252
MRPS25	71,72	100,34	1,4	0,00020709
VMA21	16,62	23,5	1,41	0,00020945
ARMCX6	52,54	74,77	1,42	5,15E-06
PAF1	54,13	76,87	1,42	7,55E-05
LDHB	1968,95	2802,46	1,42	0,0002082
MRPL40	46,64	66,59	1,43	0,00014549
MITF	13,6	19,4	1,43	0,0004074
NDUFS3	314,45	454,33	1,44	0,00010392
C20orf194	16,85	24,46	1,45	0,00037873
NNT	137,78	202,38	1,47	0,00035042
VDAC3	317,92	471,68	1,48	3,07E-05
FAM127B	103,08	154,47	1,5	0,00021944
COQ6	37,63	56,99	1,51	4,84E-05
UBE2MP1	47,65	72,14	1,51	0,00011813
NDUFAF5	55,64	83,95	1,51	0,0001269
MCAT	19,02	28,78	1,51	0,00021136
SAR1B	65,83	99,56	1,51	0,00031229
NDUFA10	151,94	231,45	1,52	0,00013586
PPP3CC	22,59	34,38	1,52	0,00024974
MRPL53	170,68	260,02	1,52	0,00038856
HINT2	63,62	97,24	1,53	6,81E-05
COA3	99,1	151,42	1,53	0,00010808
MMAB	63,07	96,2	1,53	0,00034094
COA4	75,88	117,48	1,55	6,06E-06
FAM63A	13,05	20,52	1,57	0,00013483
ALKBH1	15,05	23,81	1,58	0,00020908
AC007318.5	191,6	302,37	1,58	0,00029644
ATPAF1	102,52	162,84	1,59	4,12E-06
RP11-638I2.10	13,34	21,21	1,59	9,28E-05
NDUFB10	205,39	326,31	1,59	0,00018275
ATP5A1P3	33,54	53,35	1,59	0,0002267

UBAC1	59,03	93,94	1,59	0,00037602
FH	117,91	188,59	1,6	4,90E-05
ATP5A1	760,97	1216,89	1,6	0,00019817
PGM1	86,03	138,3	1,61	9,94E-05
ITGB1BP1	149,71	241,61	1,61	0,00010197
C7orf55	43,69	70,49	1,61	0,0003568
RP11-421N8.1	65,21	105,96	1,62	9,84E-05
C14orf2	946,3	1530,38	1,62	0,00017341
ATP5I	1301,23	2118,93	1,63	4,67E-05
NDUFA3	416,4	677,34	1,63	0,00027848
MTFR1L	131	216,43	1,65	3,08E-05
PNPO	11,59	19,16	1,65	5,45E-05
COX7A2	1129,68	1862,65	1,65	0,00011754
MRPS7	127,95	211,59	1,65	0,0003323
ELAC2	81,66	135,38	1,66	5,42E-05
MPC2	131,73	222,69	1,69	5,81E-06
ATP5F1P7	17,92	30,35	1,69	0,00019105
COX5B	418,34	708,46	1,69	0,00033955
SPRYD4	9,56	16,28	1,7	5,09E-05
IDH3B	106,48	180,64	1,7	0,00012881
MRPL2	93,91	160,04	1,7	0,00017616
PPP1R12B	63,52	108,09	1,7	0,00022949
MT-ND1	5368,64	9216,64	1,72	4,07E-05
FNDC4	10,13	17,46	1,72	5,85E-05
MXRA7	280,76	484,12	1,72	0,00018404
TACO1	24,56	42,41	1,73	2,57E-05
MT-CYB	9439,03	16367,02	1,73	9,86E-05
SDHAP1	8,15	14,15	1,74	4,72E-05
SEC61A2	20,82	36,46	1,75	0,00018614
MTATP6P1	1240,71	2168,75	1,75	0,00041824
PRKACA	152,69	268,55	1,76	1,13E-06
RPL23AP53	7,58	13,38	1,77	5,99E-05
AC007383.6	35,36	62,87	1,78	7,94E-05
AS3MT	22,06	39,55	1,79	1,36E-05
SPAG7	174,37	311,51	1,79	2,55E-05
UQCC2	199,97	358,07	1,79	0,00020508
COX10	12,38	22,18	1,79	0,0002844
TMEM176B	218,19	392,02	1,8	0,00027312
CADPS	14,14	25,41	1,8	0,00039337
MT-CO2	15998,9	29217,03	1,83	0,00015823
GRPEL1	55,39	102,48	1,85	0,00023711
TMEM246	8,34	15,44	1,85	0,00039452
DGKD	43,25	80,57	1,86	0,00021129
GBA2	72,01	134,02	1,86	0,00031326

SFXN4	59,96	113,42	1,89	0,00010535
NDUFA6	302,34	572,02	1,89	0,00013241
MFN2	118,86	224,27	1,89	0,00036342
COQ3	15,09	28,8	1,91	0,00023299
ACYP1	30,13	57,56	1,91	0,00025333
PRKAG2	138,28	265,06	1,92	1,11E-05
C10orf35	12,82	24,76	1,93	0,00010281
NDUFA7	120,33	232,29	1,93	0,00025788
ACSS2	119,85	232,49	1,94	4,46E-06
UQCRFS1	37,96	73,81	1,94	7,29E-05
PGP	14,28	27,85	1,95	4,00E-06
ZNF622	44,75	87,26	1,95	0,00021461
NEURL2	7,25	14,12	1,95	0,00023146
MRPL46	32,04	62,87	1,96	6,81E-06
VPS13D	40,87	80,31	1,97	0,00020438
PPIF	87,51	173,56	1,98	0,00039576
PGK1P2	11,85	23,5	1,98	0,00041521
HDHD1	15,21	30,25	1,99	0,00012738
SDHB	398,68	795,31	1,99	0,00024853
TMEM116	13,64	27,52	2,02	0,00016073
PRDX2	507	1030,81	2,03	0,00026883
ATP5G3	688,31	1401,91	2,04	0,00025971
ALDOAP1	21,96	45,42	2,07	6,85E-05
DCXR	196,5	408,56	2,08	6,86E-05
CS	116,84	245,74	2,1	5,40E-06
NLRP2	34,38	72,06	2,1	0,00017625
SLC25A11	102,33	214,68	2,1	0,00037598
GPI	249,43	525,79	2,11	0,00011303
CTB-52I2.4	12,33	26,08	2,12	1,95E-05
C6orf136	51,3	110,23	2,15	0,0002586
NUTM2A-AS1	5,15	11,21	2,18	0,00019484
GATB	49,74	108,85	2,19	0,00029423
CEP85	8,96	19,72	2,2	5,64E-05
SRP14-AS1	11,16	24,83	2,22	7,35E-05
FITM2	13,06	29,43	2,25	0,00030111
RILPL1	25,43	57,65	2,27	8,70E-06
ENDOG	22,17	51,25	2,31	2,75E-05
ABHD11	17,51	40,45	2,31	0,00021618
SDHA	127,59	297,51	2,33	0,00030738
COX5A	432,88	1012,9	2,34	8,37E-05
MPC1	118,13	277,08	2,35	2,82E-05
TMEM53	12,02	28,4	2,36	1,27E-06
PPP1R3C	67,49	159,5	2,36	1,31E-05
KLHL13	5,09	12,05	2,37	5,45E-06

TLE6	4,95	11,75	2,37	6,27E-05
AFAP1L1	16,55	39,32	2,38	0,00011475
FGF12	36,96	88,37	2,39	2,91E-05
STARD4	50,57	121,58	2,4	4,66E-05
FAM195A	63,46	152,64	2,41	0,0001851
GOT2	99,43	245,39	2,47	0,0002385
RP11-6N17.4	28,67	72,18	2,52	0,00042349
MIR1307	81,84	206,78	2,53	0,00029227
DUSP5	44,61	116,65	2,61	7,09E-05
COMTD1	12,01	31,5	2,62	0,00014194
PACSIN1	3,47	9,29	2,68	0,00042533
MORN4	17,7	47,74	2,7	7,51E-05
SYT11	3,15	8,57	2,72	6,71E-05
GOT1	191,25	529,11	2,77	3,58E-05
IDH3A	136,74	378,3	2,77	0,00010455
RP11-475J5.6	20,52	57,01	2,78	1,22E-05
RP11-660L16.2	6,09	17,17	2,82	2,61E-05
GADD45G	20,44	62,57	3,06	0,0004229
RAPGEF4	3,88	11,99	3,09	7,41E-05
RILP	12,31	38,2	3,1	0,00010795
PNCK	15,11	48,48	3,21	0,00016203
CHCHD10	314,08	1014,59	3,23	1,73E-05
AP000350.8	10,15	32,86	3,24	2,05E-05
EEF1A2	112,84	367,79	3,26	2,90E-05
GIPR	3,89	12,68	3,26	0,0002193
TMEM38A	6,43	21,54	3,35	2,59E-06
AP000351.3	5,25	17,64	3,36	8,94E-05
PSMC1	2,41	8,25	3,42	9,82E-07
MYOZ1	6,01	20,83	3,47	0,00019206
RPL7P3	3,82	13,59	3,56	2,07E-05
DUSP26	10,95	39,39	3,6	0,00019209
SNAP91	11,01	39,59	3,6	0,00023111
TNNI3K	21,11	80,66	3,82	0,00025841
ART5	2,06	7,92	3,84	3,93E-05
RASGRP3	9,82	38,18	3,89	4,68E-06
BDH1	11,16	43,99	3,94	2,69E-05
RP11-432J24.5	11,26	45,48	4,04	2,88E-06
SLC29A2	7,62	32,42	4,25	0,00014185
CHMP4C	3,06	12,99	4,25	0,00014614
CRYAB	1445,89	6233,3	4,31	0,00026375
NPY6R	12,59	57,99	4,61	4,16E-05
CKMT2	93,99	433,18	4,61	0,00024442
RP11-549B18.3	2,2	10,17	4,62	4,28E-05
SNORD14A	91,19	448,07	4,91	6,96E-05

ATP1A3	4,96	24,62	4,96	4,99E-05
PGAM2	49,71	260,43	5,24	6,46E-06
RP11-480I12.5	1,81	9,61	5,31	7,03E-06
RENBP	3,03	17,08	5,64	3,19E-05
SCN5A	6,34	36,78	5,8	3,22E-05
GLRXP3	0	5,66	6	7,25E-08
CKM	157,42	957,67	6,08	0,00033542
UNC13D	13,62	87,49	6,42	1,40E-05
DOK7	1,9	13,79	7,26	3,13E-05
RGN	1,33	10,18	7,65	0,00012894
RP11-264A11.1	1,35	11,86	8,79	0,00016401
SLC25A5P5	1,54	14,32	9,3	6,95E-06
DUSP13	2,62	31,09	11,87	0,00029146
RP11-350D17.3	0,77	9,39	12,19	3,76E-05
MT1G	0,39	8,08	20,72	2,87E-05
RP11-208G20.3	0	38,79	39	5,35E-14
TDGF1	0,36	21,59	59,97	1,63E-08
TDGF1P3	0,06	7,04	117,33	2,24E-07
PZP	0,34	50,99	149,97	8,15E-09
PSPHP1	0,33	81,56	247,15	2,48E-10
